# Supplementary material for: Disjoint combinations profiling (DCP): a new method for the prediction of antibody CDR conformation from sequence
Source: PeerJ. 2014 Jul 1;2:e455. doi: 10.7717/peerj.455 (PMC4103075; doi:10.7717/peerj.455)
Supplement: Supplemental Information 1 — Notable features include resolutions close to 3Å and R-free > 0.25. [file peerj-02-455-s001.doc]

| **Clusters with multi-conformation full-rogue members** | **Involved members [pdb_chain->cluster]** | **CDR sequence** | **Comments** | **Crystal notable features** |
| --- | --- | --- | --- | --- |
| L1-14-II, L1-14-VII | 1mcb_A->II, 1mcb_B->II, 1mcc_A->II, 1MCD_A->II, 1mce_A->II, 1mcf_A->II, 1MCH_A->II, 1mci_A->II, 1MCJ_A->II, 1mck_A->II, 1mcl_A->II, 1mcn_A->II, 1mcq_A->II, 1mcr_A->II, 1mcs_A->II, 1mcc_B->VII, 1mcn_B->VII, 1mcs_B->VII | TGTSSDVGGYNYVS | 1mcc: backbone fluctuation at H30aG-H30bG-H30cY due to different CDR-L3 orientation, caused by alternative binding of PEPTIDE N-ACETYL-L-GLN-D-PHE-L-HIS-D-PRO-NH2 | All structures @2.70Å resolution. R-free: n/a |
| L1-14-III, L1-14-IV | 1jvk_B->III, 1lgv_B->III, 1lhz_B->III, 1jvk_A->IV, 1lgv_A->IV, 1lhz_A->IV | TGVSSIVGSYNLVS | L27-L30b backbone fluctuation due to asymmetrical Bence-Jones dimer binding (slightly dislocated CDR-L3), non-intruding crystal symmetry. | 1jvk: R-free = 0.254 |
| L3-8-I, L3-8-V | 1fn4_A->I, 1fn4_C->I, 1F3R_B->V | YQYNNGYT | 1f3r: bound to ACETYLCHOLINE RECEPTOR ALPHA | 1fn4: Resol. = 2.80Å, R-free = 0.294 1F3R: NMR structure |
| L3-10-II, L3-10-XII | 1a8j_L->II, 2mcg_2->II, 1a8j_H->XII, 2mcg_1->XII | SSYEGSDNFV | Asymmetrical light dimer binding | 1a8j: Resol. = 2.70Å, R-free = 0.38 2mcg: R-free: n/a |
| L3-10-II, L3-10-III | 1keg_L->II, 1ehl_L->III | FQGSLVPT | Different DNA fragment bound | 1keg, 1ehl: R-free > 0,25 |
| L3-10-II, L3-10-V | 3DGG_C->II, 3DGG_A->V | QHSRELLT | Backbone flip at L92R due to small discrepancies in the mirror-image binding of the light chains from the 2 identical fabs. | - |

**Supplementary Table:** Detected multi-conformation full-rogue clusters, for Light chain CDRs. Notable features include resolutions close to 3Å and R-free > 0.25.

| **Clusters with multi-conformation full-rogue members** | **Involved members [pdb_chain->cluster]** | **CDR sequence** | **Comments** | **Crystal notable features** |
| --- | --- | --- | --- | --- |
| L3-10-II, L3-10-VII, L3-10-XII | 1mcb_A->II, 1mcc_A->II, 1MCD_A->II, 1mce_A->II, 1mcf_A->II, 1MCH_A->II, 1mci_A->II, 1MCJ_A->II, 1mck_A->II, 1mcl_A->II, 1mcn_A->II, 1mcq_A->II, 1mcr_A->II, 1mcf_B->II, 1mcs_A->VII, 1mcb_B->XII, 1mcc_B->XII, 1MCD_B->XII, 1mce_B->XII, 1MCH_B->XII, 1mci_B->XII, 1MCJ_B->XII, 1mck_B->XII, 1mcl_B->XII, 1mcn_B->XII, 1mcq_B->XII, 1mcr_B->XII, 1mcs_B->XII | SSYEGSDNFV | Asymmetrical light dimer binding | All structures @2.70Å resolution. R-free: n/a, or better than 0.25 |
| L3-10-II, L3-10-VII | 3mcg_2->II, 1mcw_M->VII | SSYEGSDNFV | 3mcg: light dimer (MCG-MCG), 1mcw: hybrid dimer (WEIR-MCG) | 1mcw: Resol. = 3,50Å, R-free: n/a, 3mcg: R-free: n/a |
| L3-10-II, L3-10-XII | 1lil_B->II, 1lil_A->XII | QVWDSNASVV | backbone fluctuation at L93S-L94N-L95A - no apparent reason other than backbone flexibility (also, different L2Y positioning) | 1lil: Resol. = 2.65Å |
| L3-11-I, L3-11-V | 1bjm_B->I, 4bjl_B->I, 4bjl_A->V | AAWDDSLDVAV | Backbone fluctuation at L94-L95-L95a - possible backbone flexibility | 1bjm, 4bjl: R-free: n/a |

**Supplementary Table:** Detected multi-conformation full-rogue clusters, for Light chain CDRs.

| **Clusters with multi-conformation full-rogue members** | **Involved members [pdb_chain->cluster]** | **CDR sequence** | **Comments** | **Crystal notable features** |
| --- | --- | --- | --- | --- |
| H1-13-I, H1-13-XI | 1n7m_L->I, 1ngz_B->I, 1ngx_B->XI, 1ngx_H->XI | KASGYTFTSYWMH | 1ngx bound to JEF ligand | 1n7m, 1ngx: R-free >= 0.25 |
| H1-13-II, H1-13-IV | 3QXW_D->II, 3QXV_A->IV, 3QXV_B->IV, 3QXV_C->IV, 3QXV_D->IV, 3QXV_E->IV | AASRRSSRSWAMA | 3QXV bound to MTX/SO4 | 3qxv: R-free = 0.279 |
| H2-9-I, H2-9-VI | 2aj3_B->I, 2aj3_D->VI, 2aj3_F->VI | NVYDSGDTN | 2aj3_B: increased solvent accessibility | 2aj3:  Resol. = 2.03Å, R-free = 0.269 |
| H2-10-II, H2-10-V | 1G9E_A_13->II, 1G9E_A_4->V | AINWDSARTY | Backbone fluctuation at H54-H55 *(possible flexibility)* | 1g9e: NMR structure |
| H2-10-II, H2-10-VI | 2P42_B->II, 2P42_D->II, 2P43_B->II, 2P49_B->II, 1bzq_K->VI, 1bzq_L->VI, 1bzq_M->VI, 1bzq_N->VI | AMDSGGGGTL | 1bzq: Backbone fluctuation @ H54G (poly-G) + antigen presence (RNASE A) | 1bzq: Resol. = 2.80Å, R-free = 0.282 |
| H2-10-II, H2-10-IX | 2fjf_H->II, 2fjf_B->II, 2fjf_D->II, 2fjf_F->II, 2fjf_I->II, 2fjf_K->II, 2fjf_N->II, 2fjf_P->II, 2fjf_R->II, 2fjf_T->II, 2fjf_V->II, 2fjf_X->II, 2fjg_H->IX, 2fjg_B->IX | GITPAGGYTY | 2fjg: bound to Vascular Endothelial Growth Factor A | 2fjf: Resol. = 2.65Å, 2fjg: Resol. = 2.80Å |
| H3-5-I, H3-5-IV | 1n7m_L->I, 1ngx_B->IV, 1ngx_H->IV | RDSDY | 1n7m: bound to MMP 1ngx: bound to JEF | 1n7m, 1ngx: R-free >= 0.25 |
| H3-6-I, H3-6-II | 2EH8_H->I, 2EH7_H->II | EYDEAY | 2EH8: bound to PreS1/PreS2/surface protein, light chain dislocation | 2eh7, 2eh8: R-free > 0.25 |

**Supplementary Table:** Detected multi-conformation full-rogue clusters, for Heavy chain CDRs.

| **Clusters with multi-conformation full-rogue members** | **Involved members [pdb_chain->cluster]** | **CDR sequence** | **Comments** | **Crystal notable features** |
| --- | --- | --- | --- | --- |
| H3-9-III, H3-9-IV | 1igt_D->III, 1igt_B->IV | HGGYYAMDY | Minor fluctuation around H96G, H97G | 1igt: Resol. = 2.80Å, R-free = 0.297 |
| H3-10-VI, H3-10-XXVIII | 2gfb_B->VI, 2gfb_D->VI, 2gfb_F->VI, 2gfb_H->VI, 2gfb_J->VI, 2gfb_L->VI, 2gfb_N->VI, 2gfb_P->VI, 1kno_B->XXVIII, 1kno_D->XXVIII, 1kno_F->XXVIII | GDYYGSRGAY | 2gfb: packed against Heavy Fab + low resolution in both 2gfb/1kno. Possible crystallogra phic error. | 2gfb: Resol. = 3.00Å, R-free: n/a, 1kno: Resol. = 3.20Å, R-free = 0.272 |
| H3-13-VI, H3-13-XX | 2GK0_H->VI, 2GJZ_H->XX, 2GJZ_B->XX | HWGGYYIPYGMDH | Backbone fluctuation at H98G *(flexibility)* | 2gk0, 2gjz: R-free >= 0.25 |
| H3-14-II, H3-14-X | 1op3_H->II, 1op3_M->II, 1op5_H->II, 1op5_M->II, 1zls_H->II, 1zlu_H->II, 1zlu_M->II, 1zlv_M->II, 1zlw_H->II, 1zlw_M->II, 2OQJ_B->II, 2OQJ_E->II, 2OQJ_H->II, 2OQJ_K->II, 1om3_K->X | KGSDRLSDNDPFDA | 1op3: bound to Mannose | Best resolutions per conformation: 1op3: 1.75Å (R-free = 0.251), 1om3: 2.20Å (R-free = 0.266) |
| H3-15-II, H3-15-XIV | 3MLR_H->II, 3MLS_H->II, 3MLS_I->II, 3MLS_J->II, 3MLS_K->II, 3MLT_H->II, 3MLT_B->II, 3MLT_E->XIV, 3MLT_I->XIV | LYLFEGAQSSNAFDL | 3MLR, 3MLS, 3MLT_H/B: bound to HIV-1 gp120 V3, light chain dislocation from antigen. 3MLT_E/I, not bound. | 3MLS, 3MLT: R-free > 0.25 |
| H3-15-V, H3-15-VII | 2VYR_I->V, 2VYR_J->V, 2VYR_K->V, 2VYR_L->V, 2VYR_E->VII, 2VYR_F->VII, 2VYR_G->VII, 2VYR_H->VII | PWYPFMASKGSEFDY | 2VYR_E/F/G/H: bound to MDM4 | - |
| H3-16-II, H3-16-V | 3CSY_A->II, 3CSY_C->II, 3CSY_E->II, 3CSY_G->II, 3INU_H->V, 3INU_M->V | EGPRATGYSMADVFDI | 3CSY: bound to Envelope Glycoprotein GP1+GP2 (H100d:MSE) | 3CSY: Resol. = 3.40Å, R-free = 0.302, 3INU: R-free = 0.252 |

**Supplementary Table:** Detected multi-conformation full-rogue clusters, for Heavy chain CDRs.

| **Different CDR-H3-base conformations displayed by identical rogue sequences** | **Involved members [pdb_chain->baseType]** | **Comments** | **Crystal notable features** |
| --- | --- | --- | --- |
| Kinked(I)/Extended Negative(II) | 1q9q_B->I, 1q9r_B->I, 1q9t_B->I, 1q9v_B->I, 2R1W_B->I, 2R1X_B->I, 2R1Y_B->I, 2R23_B->I, 2R2B_B->I, 2R2E_B->I, 2R2H_B->I, 3BPC_B->I, 3SY0_B->I, 3T4Y_B->I, 3T65_B->I, 3T77_B->I, 1q9l_D->II | 1q9l unliganded | 1q9l: Resol. = 2.28Å, R-free = 0.286 |
| Kinked(I)/Extended Negative(II) | 1F3R_B->I, 1fn4_B->II, 1fn4_D->II | 1fn4: malformation of CDR-H3, HFR3 displacement. | 1f3r: NMR structure, 1fn4: Resol. = 2.80Å, R-free = 0.294 |
| Extended Negative(II)/ Extended Positive(III) | 3C08_H->II, 3C09_H->III, 3C09_C->III | 3C09 liganded, low resolution. | 3C09: Resol. = 3.20Å, R-free = 0.299, 3C08: R-free = 0.264 |
| Kinked(I)/Extended Negative(II) | 1oaq_H->I, 1oar_H->I, 1oar_I->I, 1oar_J->I, 1oar_K->I, 1oau_H->I, 1oau_J->I, 1oaz_H->I, 1oaz_J->I, 1ocw_H->II | 1ocw: possible invasive crystal packing. | 1oaq: R-free = 0.181, 1ocw: R-free = 0.29 |
| Kinked(I)/Extended Positive(III) | 3OKD_B->I, 3OKE_B->I, 3OKK_B->I, 3OKL_B->I, 3OKN_B->I, 3OKO_B->I, 3OKM_B->III | Only 3OKM is ligand free. Other structures contain Kdo/Ko interacting with the CDR-H3 apex. | R-free > 0.25: 3OKE(0.289), 3OKD(0.275), 3OKM(0.306), 3OKN(0.269), 3OKO(0.267) |

**Supplementary Table:** Structures with identical Fv sequences, displaying different CDR-H3-base conformation.
