# Supplementary material for: Disjoint combinations profiling (DCP): a new method for the prediction of antibody CDR conformation from sequence
Source: PeerJ. 2014 Jul 1;2:e455. doi: 10.7717/peerj.455 (PMC4103075; doi:10.7717/peerj.455)
Supplement: Supplemental Information 2 — Presentation of a probabilistic closed-form equation for selecting only statistically significant signature signals. [file peerj-02-455-s002.doc]

**Appendix: absolute probabilistic significance of IF fragment disjointness**

Consider whether the observation of disjointness between a number n of Target- and a number m of Query-fragments, was by itself an event of statistical significance. This problem can be translated into an urn-and-ball analogy: there are two urns A and B, each containing theoretically the same number N of number-labelled balls, where N equals the number of possible sequence combinations that can be produced by 20 amino-acids in each IF fragment position. For example, when considering couplets, then each urn contains 20*20=400 labelled balls, one for each sequence combination; 20*20*20=8000 labelled balls for triplets, *etc*. Making m draws from urn A and n draws from urn B (with replacement) resulted in disjoint sets of outcomes between n and m. Therefore, the likelihood that urns A and B contain all the possible labelled balls in equal numbers (the null hypothesis) against a hypothesis that the urns actually contain disjoint sets of labels/numbers, needs to be assessed. In other words there is the need to calculate whether the produced disjoint outcome can be attributed to chance, or if it could be pointing to a preferential, exclusive and conformation-influencing, selection of residues by the members of each class.

The above probability was called P_disjoint_(N,m,n), with N=20 for singletons, N=400 for couplets, N=8000 for triplets, N=160000 for quadruplets, *etc*. The calculation of P_disjoint_(N,m,n) is done by summing the probabilities over all possible numbers of unique outcomes r:

$P_{disjo\mathrm{int}}(N,m,n)=\frac{\sum_{r=0}^{N} \left(\begin{matrix} N \\ r \end{matrix}\right)r!\left\{\begin{matrix} m \\ r \end{matrix}\right\}(N-r)^{n}}{N^{m+n}}$ (I)

Where:

$\left(\begin{matrix} N \\ r \end{matrix}\right)=\frac{N!}{r!(N-r)!}$, is the number of ways to choose r of N ball numbers to be the outcomes of the drawings from urn A.

$\left\{\begin{matrix} m \\ r \end{matrix}\right\}=\frac{1}{r!}\sum_{k=0}^{r} (-1)^{r-k}\left(\begin{matrix} r \\ k \end{matrix}\right)k^{m}$, is a Stirling number of the second kind, which counts the number of ways that the m outcomes can take r chosen values assuring that each value is chosen at least once. This number is multiplied by r!, because the order of the outcomes matters (ordered Bell number).

(N-r)^n^, is the number of ways to choose the n balls from urn B so that they are disjoint from the numbers drawn from urn A.

Finally, their sum is divided by N^m+n^, which is the number of ways to choose m balls from urn A and n balls from urn B, without regard for disjointness.

This probability was calculated on every training occasion between m Target IF sequences and n Query IF sequences. Theoretically, with P_disjoint_<0.05, or more strictly, with P_disjoint_<0.01 or even P_disjoint_<0.001, the null hypothesis can be rejected and all IF fragments of the selected order (*e.g.* couplets, N=400, triplets, N=8000, *etc*.) can be retained as significant and potentially conformation-influencing, when comparing sequences corresponding to different conformations. In practice, the null hypothesis was most easily rejectable for couplets only; however disjoint IF couplet fragments were seldom detected throughout the comparisons performed during training. On the other hand, for the frequently detected disjoint IF triplet fragments, P_disjoint_ could rarely reach a value of significance over all clusters of a CDR/Length, due to the uneven stratification of cluster populations. For these reasons, this calculation of absolute probabilistic significance for disjointness could not be employed as a global filtering criterion for signature signals. However, it is provided here as a tool that can potentially lead to more specific DCP signatures when cluster populations become richer. Alternatively, equation (I) could be combined with the use of reduced sets of non-overlapping amino acid alphabets, in which case number N can be greatly reduced and therefore increase the potential of statistical validation of acquired signature signals.

Below are several numerical examples derived directly from the training procedure, provided only for appreciation of the order of statistical significance of signature signals per cluster/per combinatorial order:

*CDR-L1/11-residues: 4 clusters*

*L1-11-I training, Target: 19, Query: 261*

*Singletons:*

*P_disjoint_ (20, 19, 261) = 2.7*10^-29^*

*Couplets:*

*P_disjoint_ (400, 19, 261) = 4.5*10^-6^*

*Triplets:*

*P_disjoint_ (8000, 19, 261) = 0.54*

*L1-11-II training, Target: 270, Query: 10*

*Singletons:*

*P_disjoint_ (20, 270, 10) = 1.9*10^-18^*

*Couplets:*

*P_disjoint_ (400, 270, 10) = 0.0012*

*Triplets:*

*P_disjoint_ (8000, 270, 10) = 0.71*

*CDR-H2/10-residues: 10 clusters*

*H2-10-I training, Target: 124, Query: 198*

*Singletons:*

*P_disjoint_ (20, 124, 198) = 9.5*10^-89^*

*Couplets:*

*P_disjoint_ (400, 124, 198) = 3.0*10^-26^*

*Triplets:*

*P_disjoint_ (8000, 124, 198) = 0.046*

*H2-10-VII training, Target: 320, Query: 2*

*Singletons:*

*P_disjoint_ (20, 320, 2) = 3.7*10^-9^*

*Couplets:*

*P_disjoint_ (400, 320, 2) = 0.20*

*Triplets:*

*P_disjoint_ (8000, 320, 2) = 0.92*
