# Supplementary material for: Disjoint combinations profiling (DCP): a new method for the prediction of antibody CDR conformation from sequence
Source: PeerJ. 2014 Jul 1;2:e455. doi: 10.7717/peerj.455 (PMC4103075; doi:10.7717/peerj.455)
Supplement: Supplemental Information 4 — Sequence logos were constructed for the training clusters using Berkeley’s WebLogo facility (http://weblogo.berkeley.edu/logo.cgi). [file peerj-02-455-s004.pdf]

H1-10-I

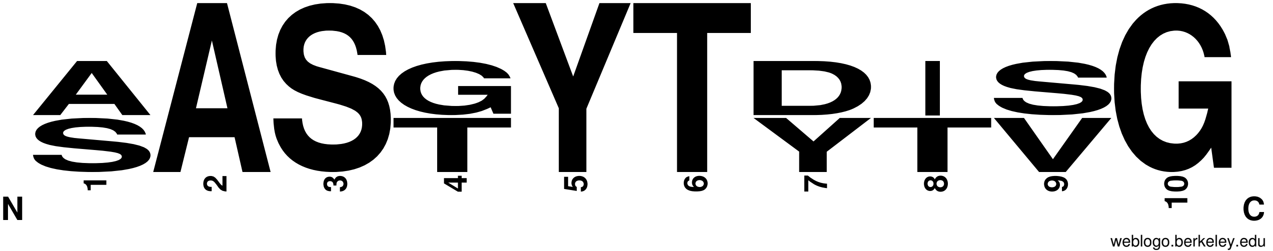

H1-13-I

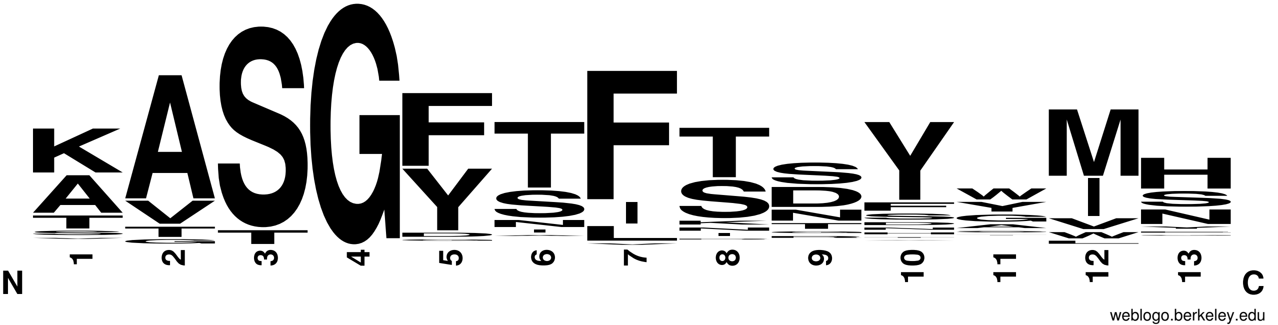

H1-13-II

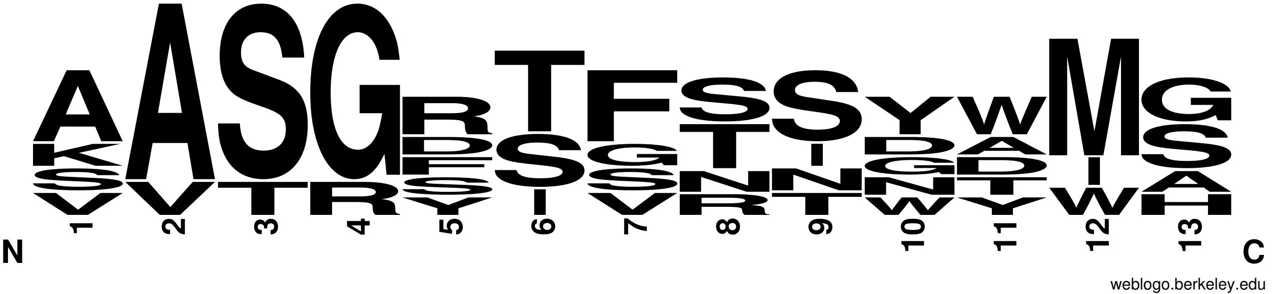

H1-13-III

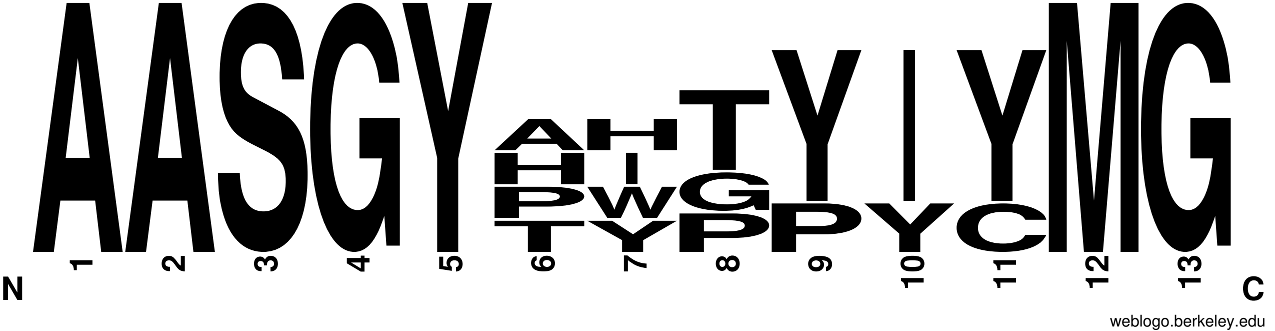

H1-13-IV

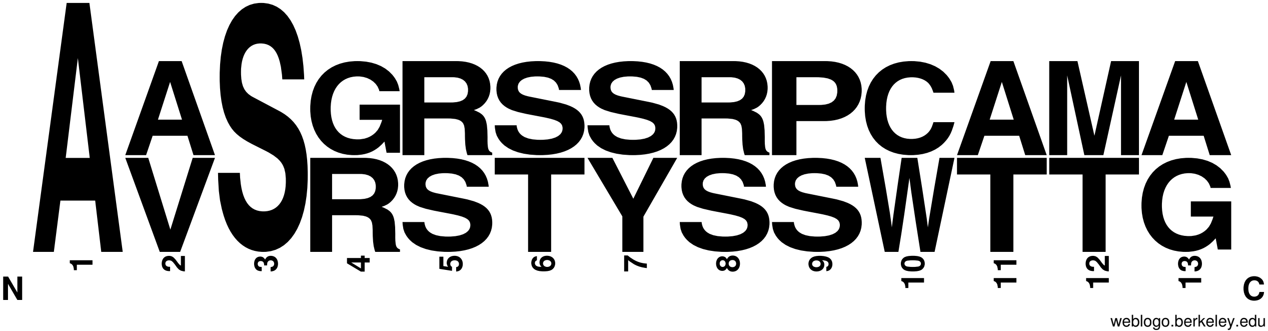

H1-13-V

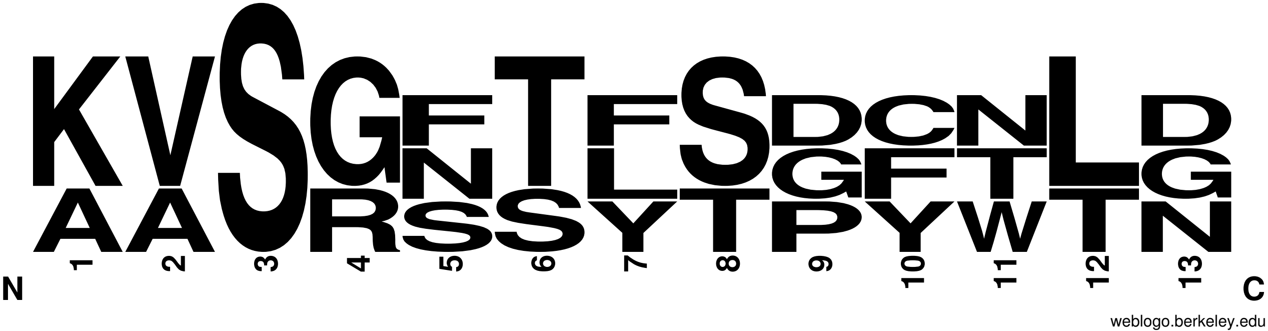

H1-13-VI

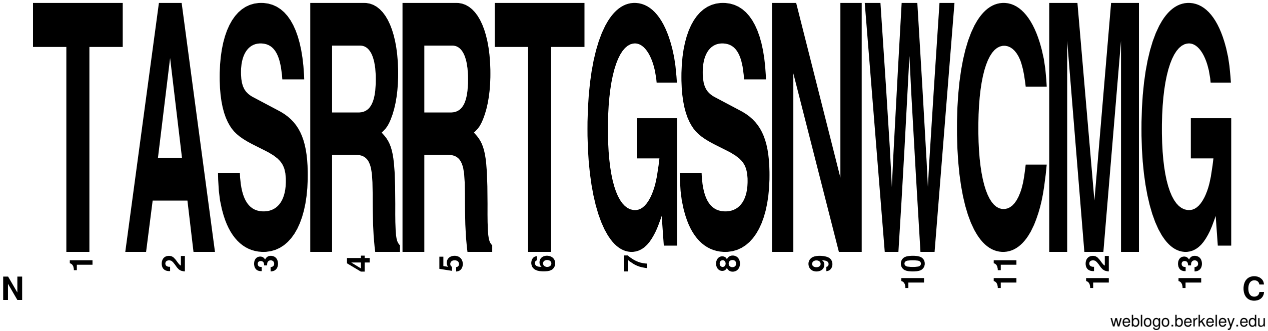
TASRRTGSNWCMG
1 2 3 4 5 6 7 8 9 10 11 12 13
N
C
weblogo.berkeley.edu

H1-13-VII

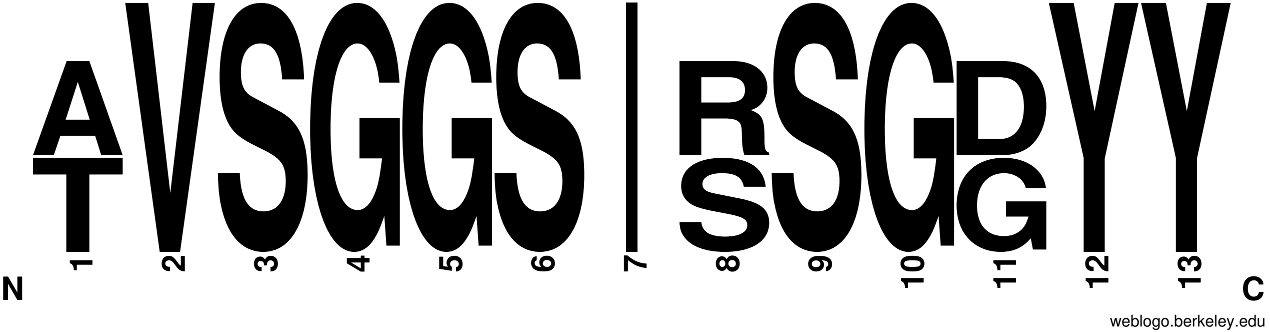

H1-13-VIII

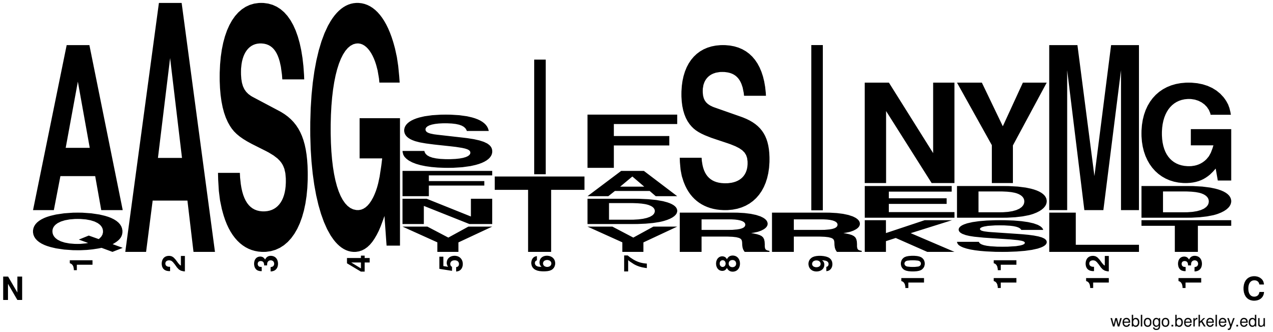

H1-13-IX

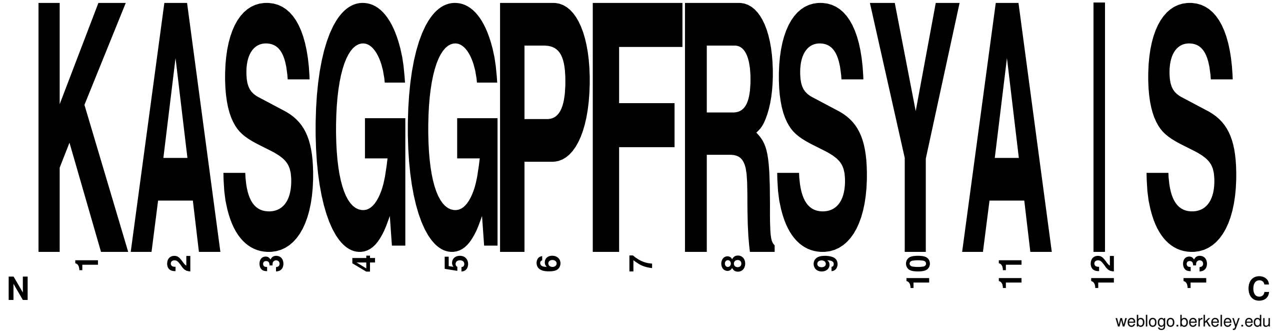
KASGGPFRSYAIS
1
2
3
4
5
6
7
8
9
10
11
12
13
N
C
weblogo.berkeley.edu

H1-13-X

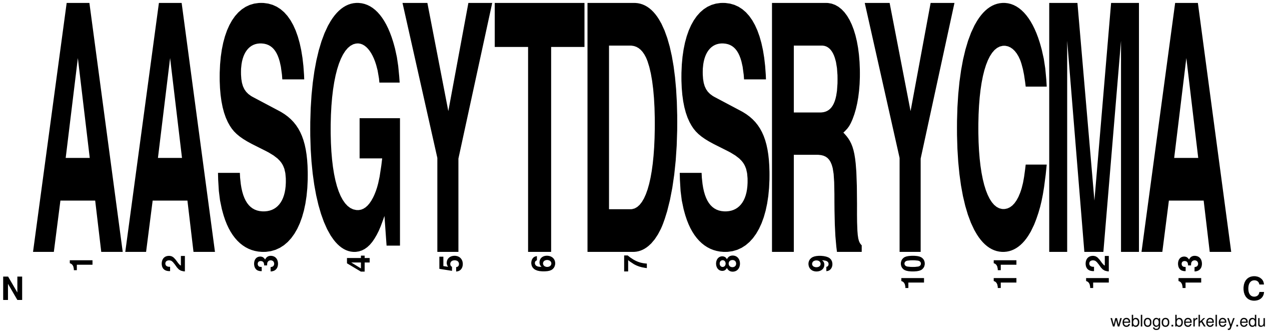

AASGYTDSRYCMA
1 2 3 4 5 6 7 8 9 10 11 12 13
N
C
weblogo.berkeley.edu

H1-13-XI

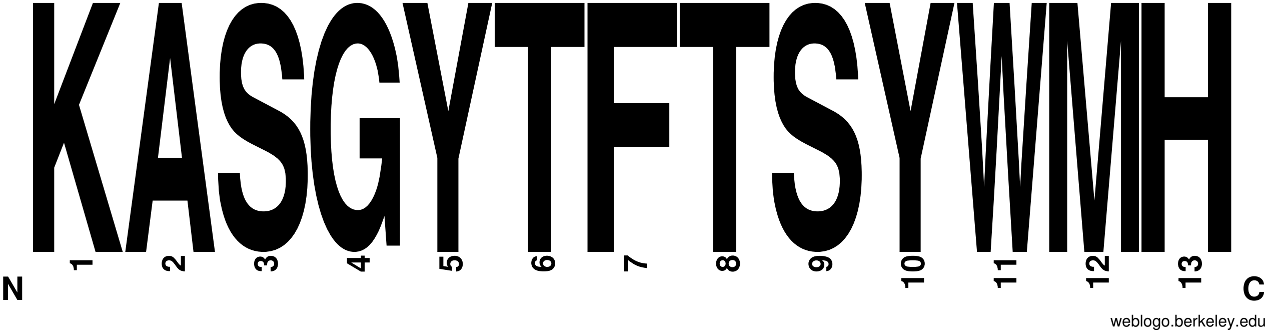
KASGYTFTSYWMH
N
1 2 3 4 5 6 7 8 9 10 11 12 13
C
weblogo.berkeley.edu

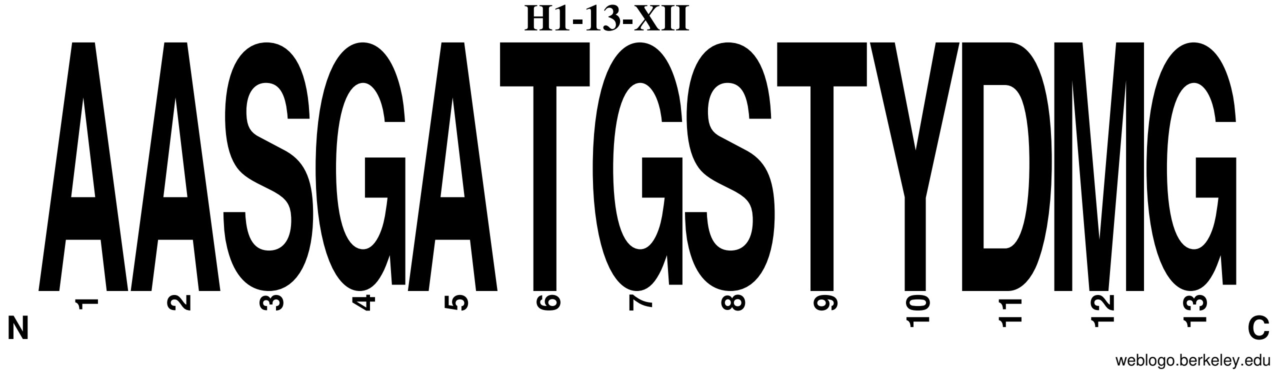
H1-13-XII
AASGATGSTYDMG
N
1 2 3 4 5 6 7 8 9 10 11 12 13
C
weblogo.berkeley.edu

H1-14-I

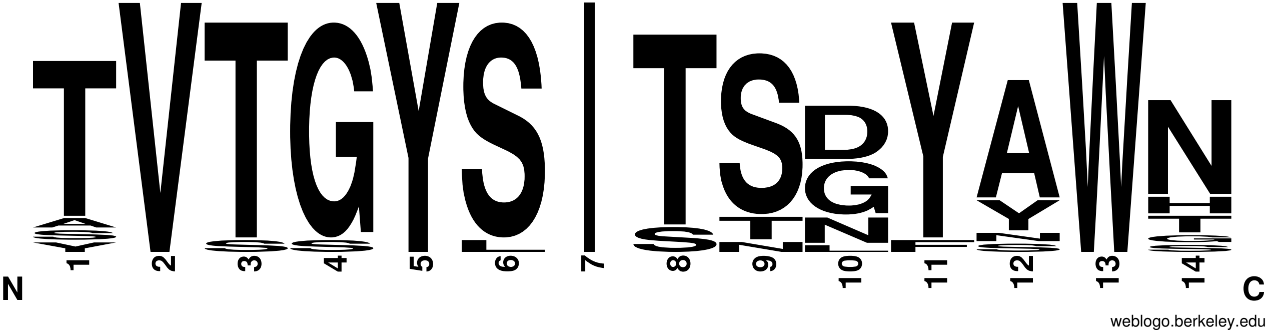

H1-15-I

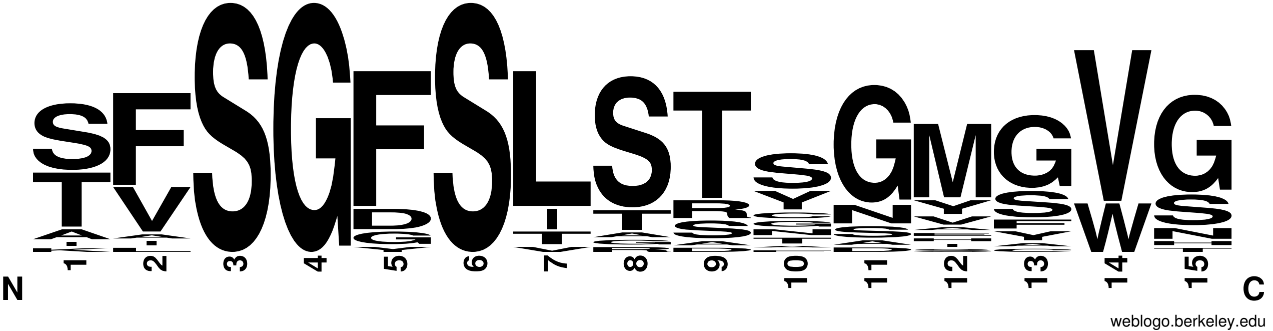

H1-15-II

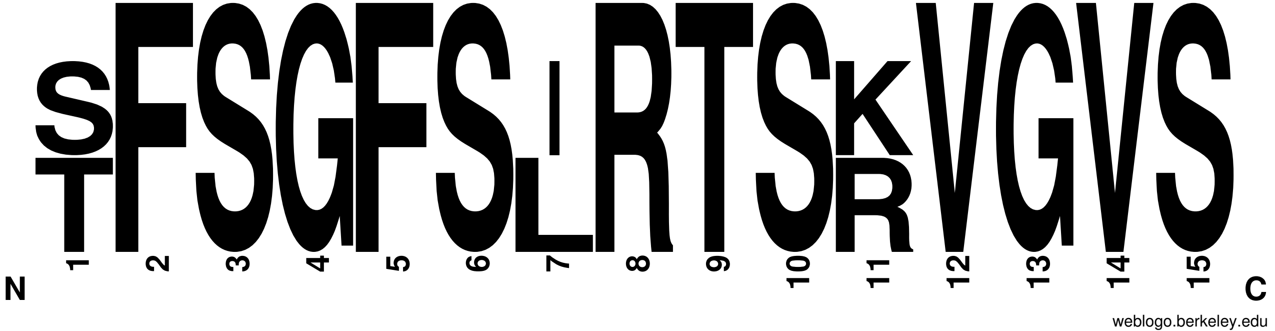

H1-15-III

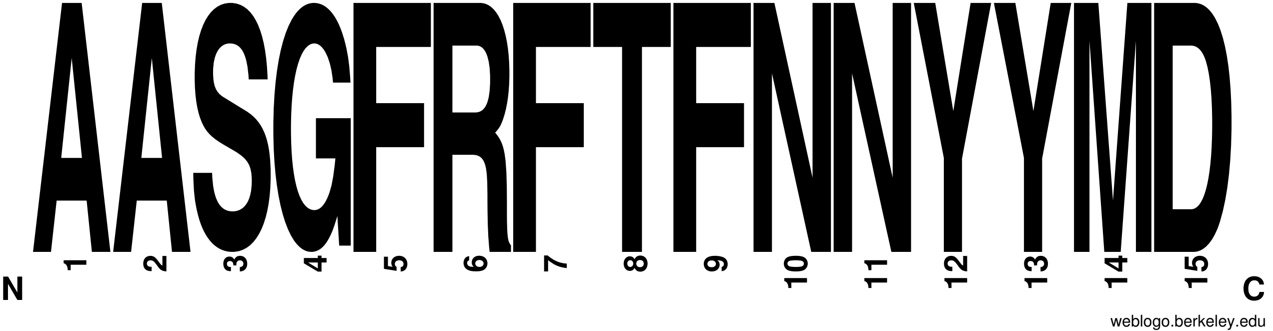
AASGFRFTFNNYYMD
1 2 3 4 5 6 7 8 9 10 11 12 13 14 15
N
C
weblogo.berkeley.edu

H1-16-I

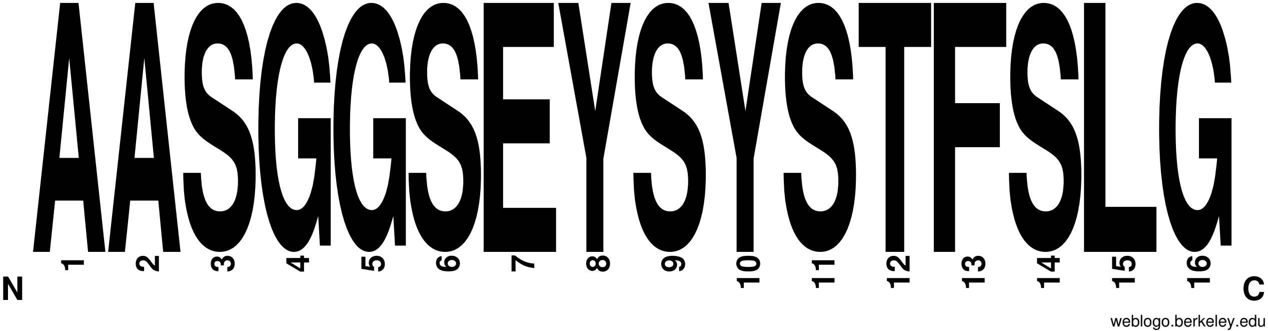
AASGGSEYSYSTFSLG
N
1 2 3 4 5 6 7 8 9 10 11 12 13 14 15 16
C
weblogo.berkeley.edu

H2-8-I

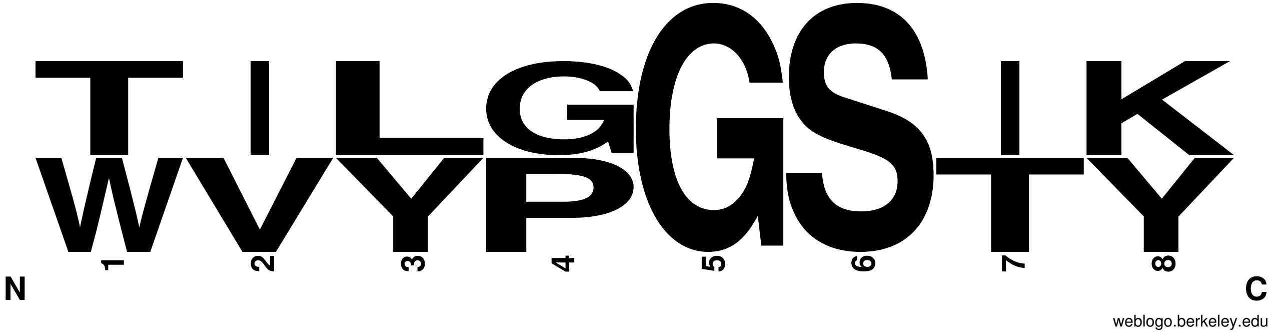

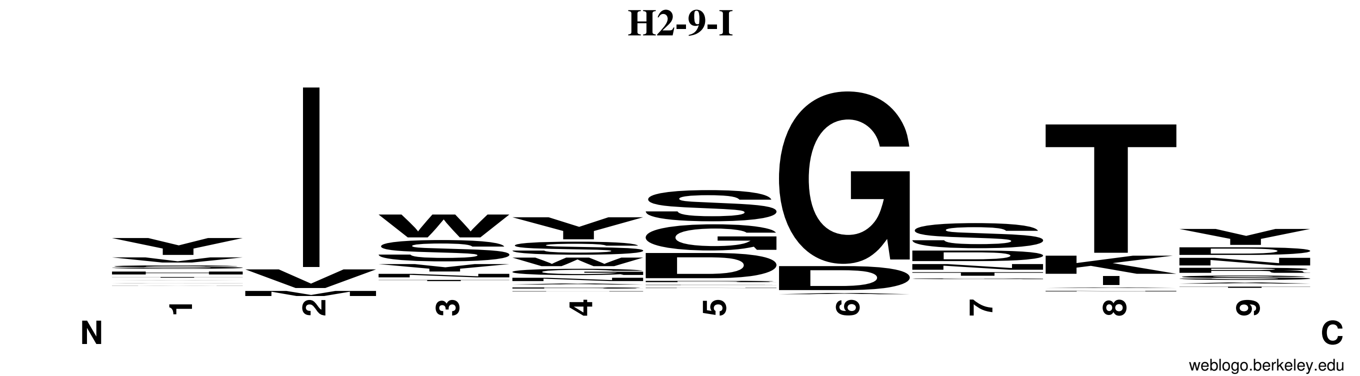
H2-9-I
1
2
3
4
5
6
7
8
9
N
C
weblogo.berkeley.edu

H2-9-II

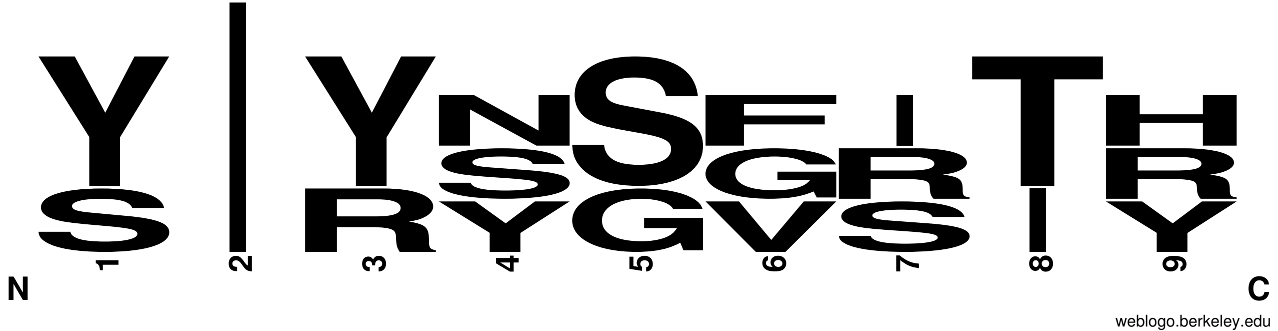

H2-9-III

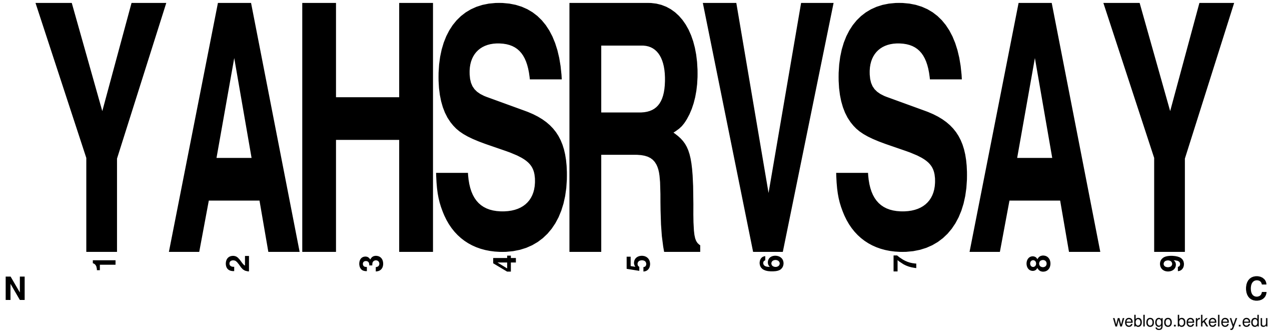

H2-9-IV

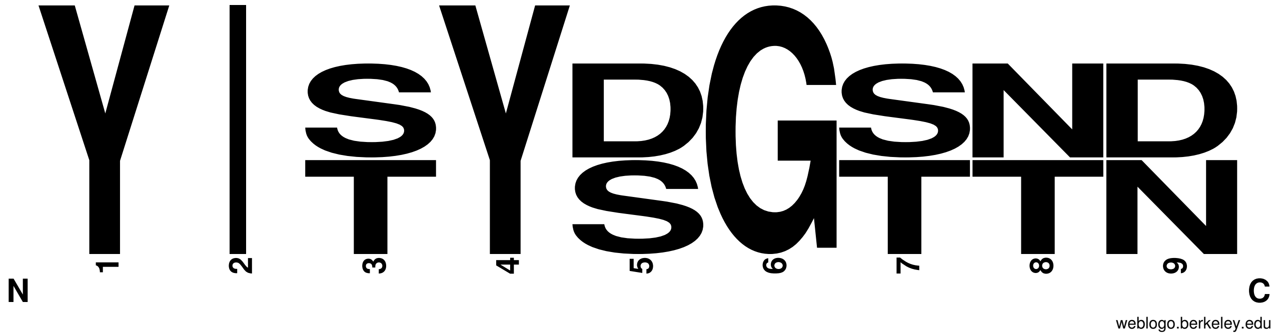

H2-9-V

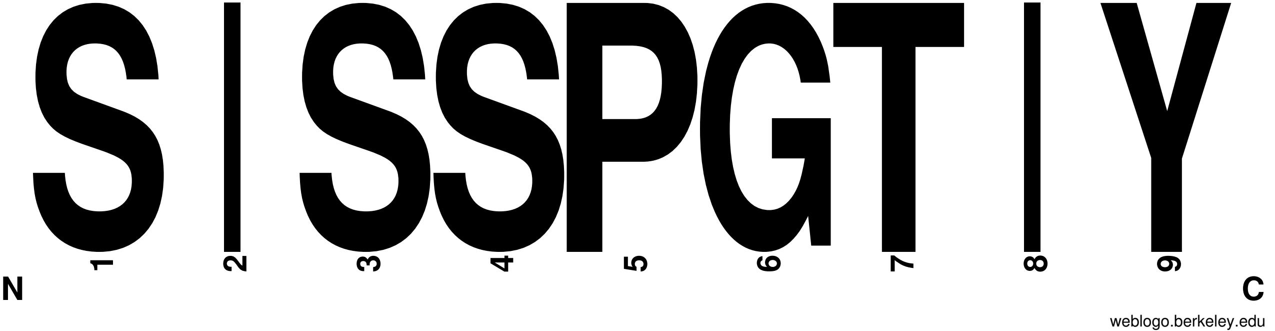

H2-9-VI

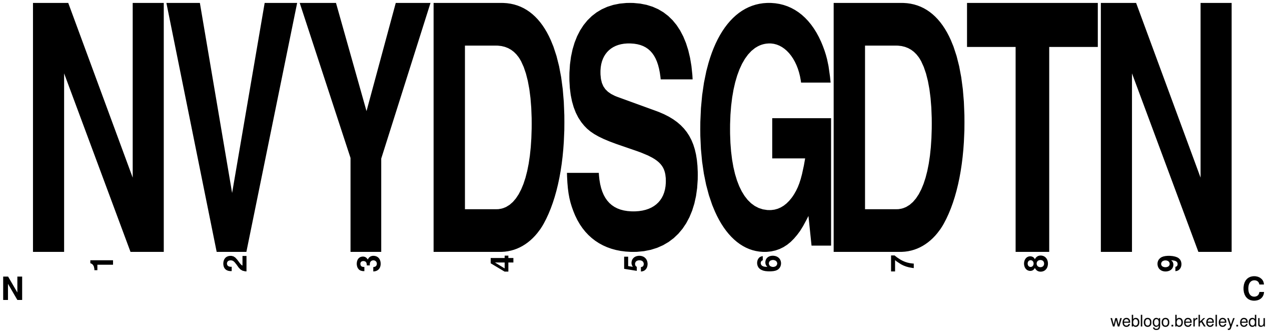

H2-10-I

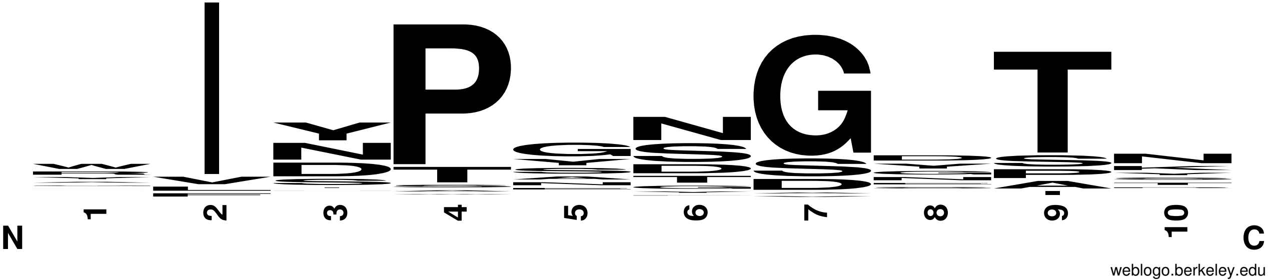

H2-10-II

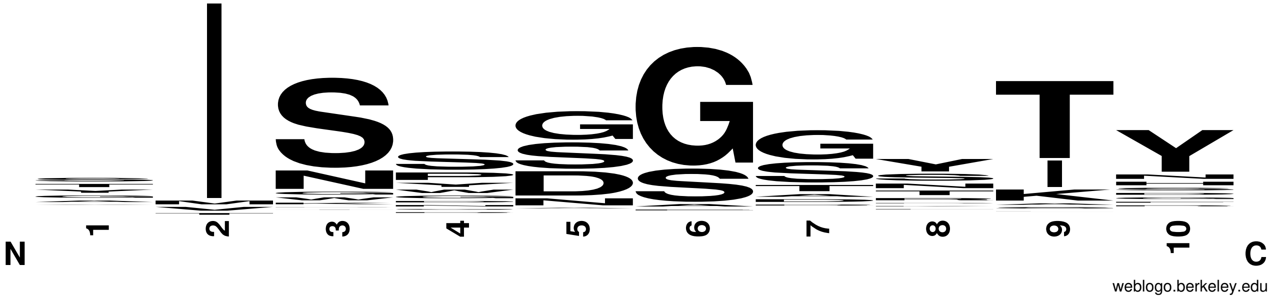

H2-10-III

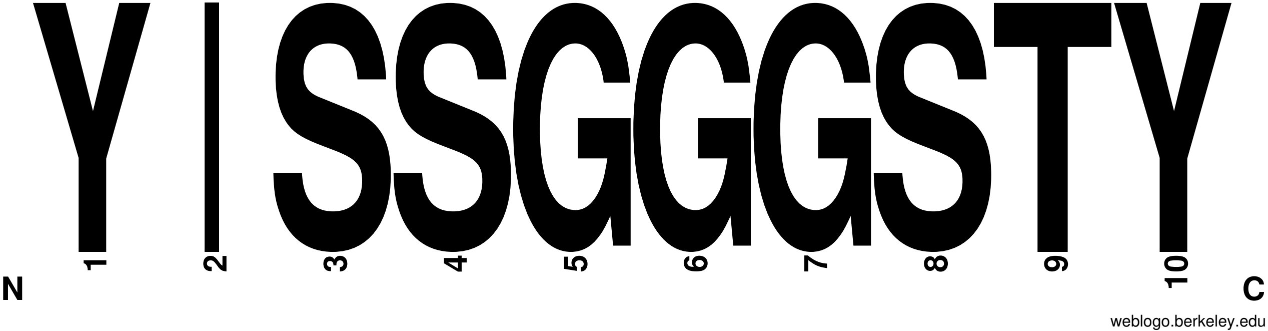

H2-10-IV

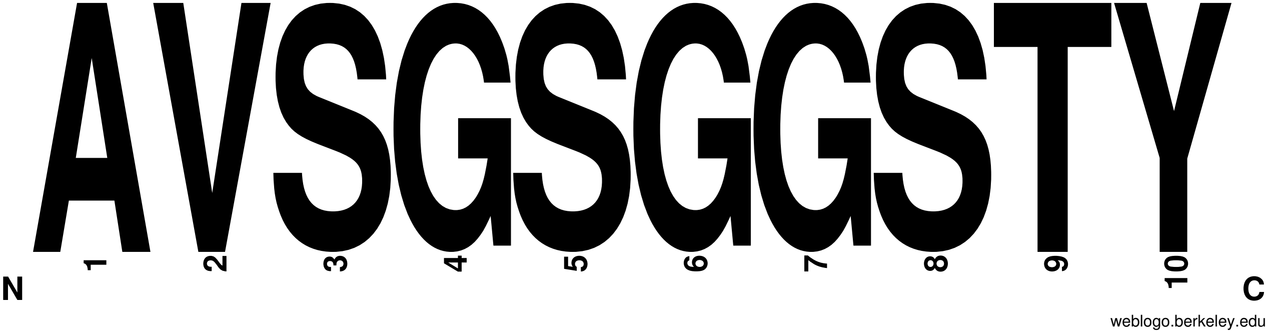

H2-10-V

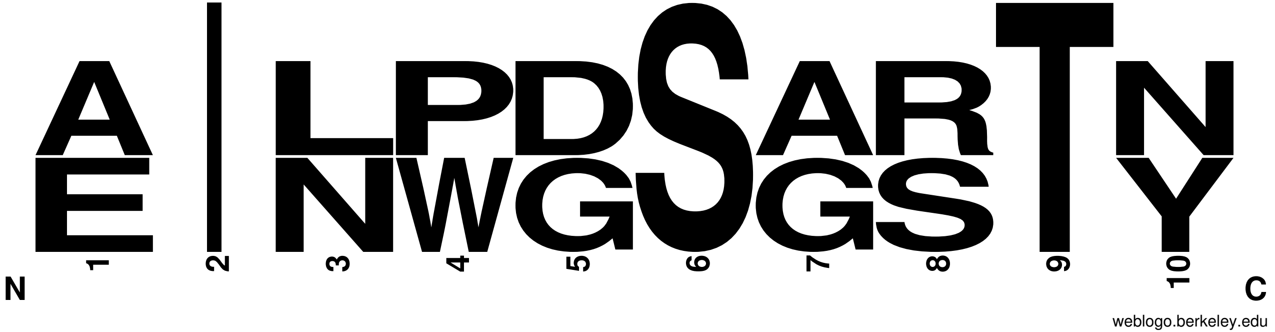

H2-10-VI

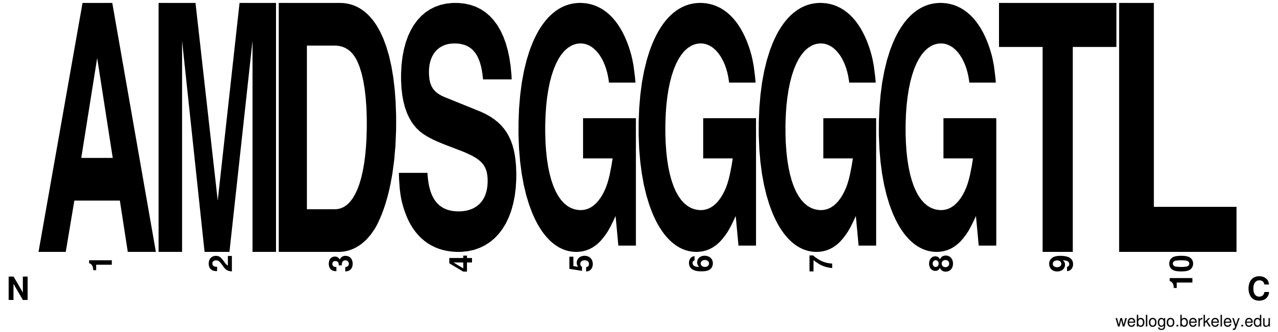

H2-10-VII

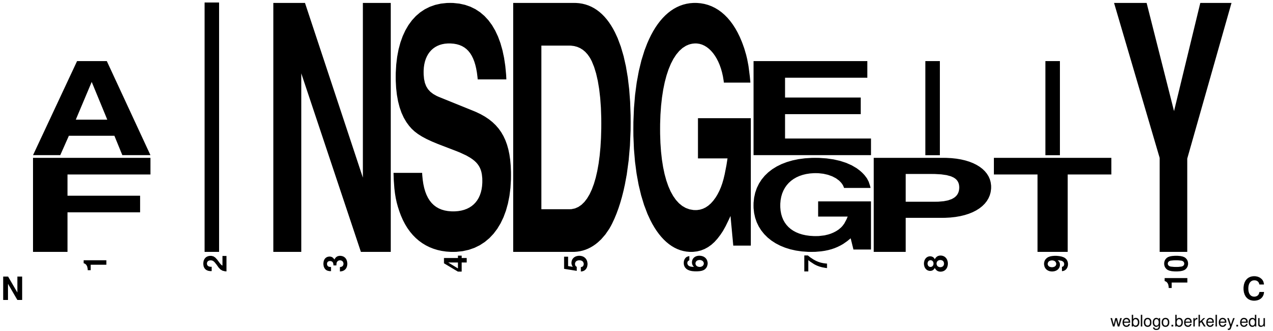

H2-10-VIII

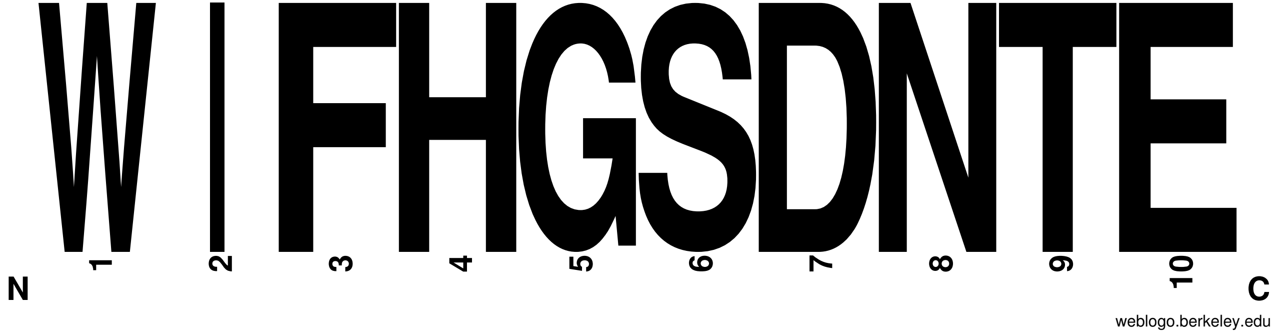

H2-10-IX

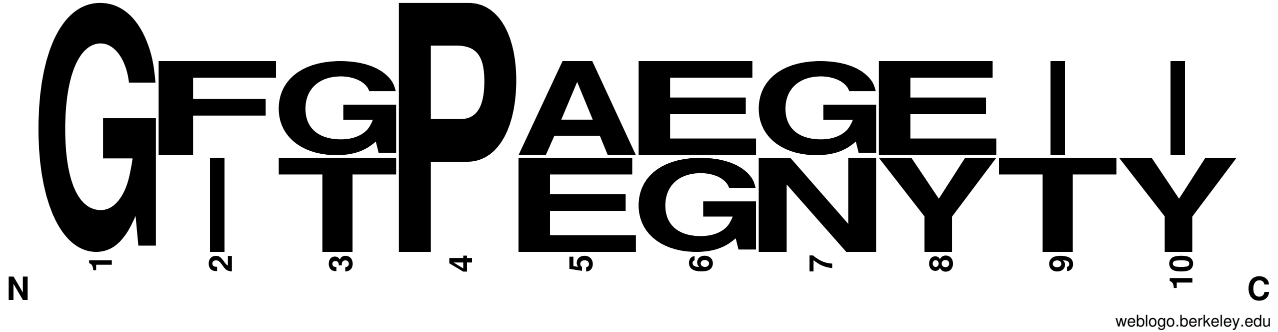

H2-10-X

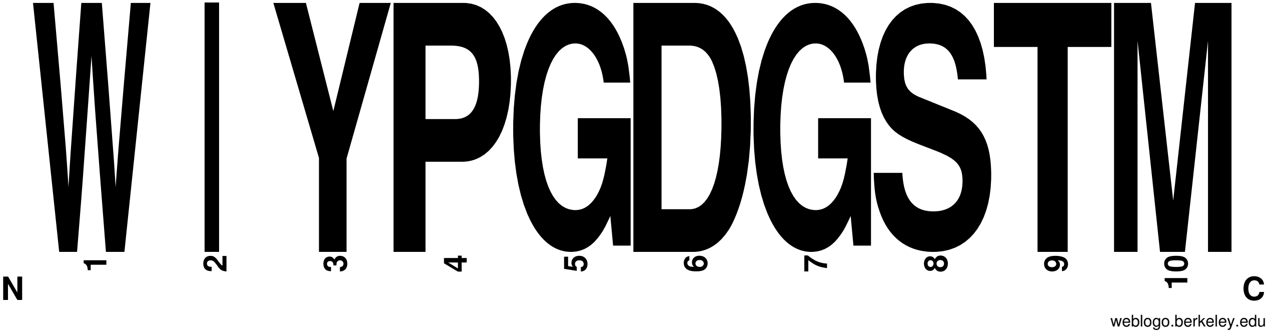

H2-11-I

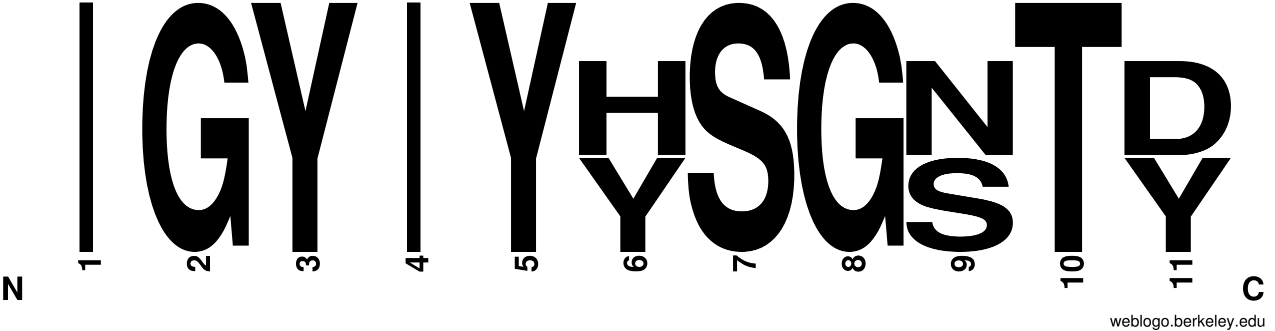

H2-12-I

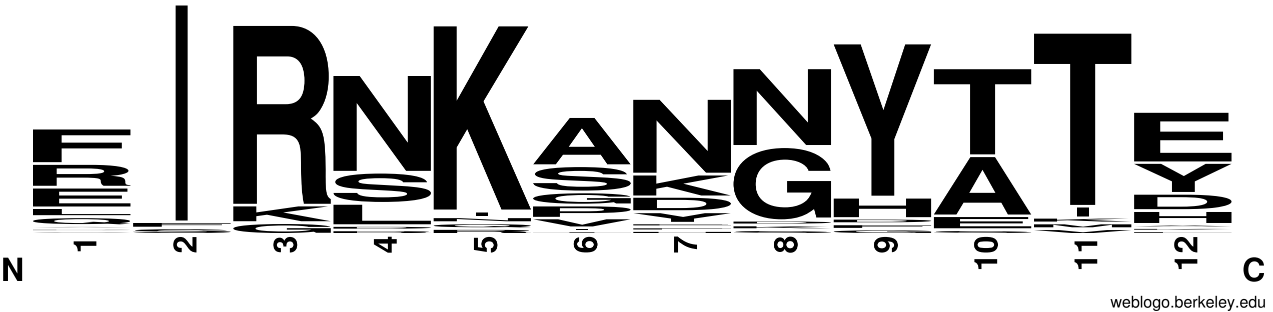

H2-12-II

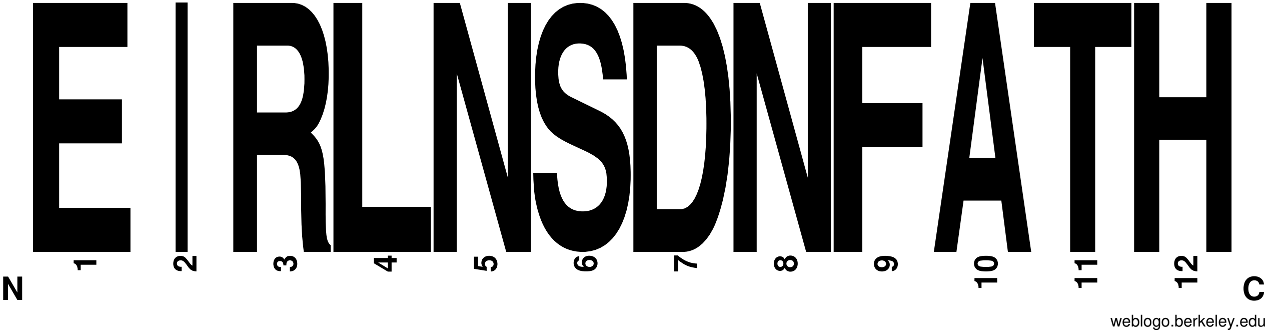

H2-12-III

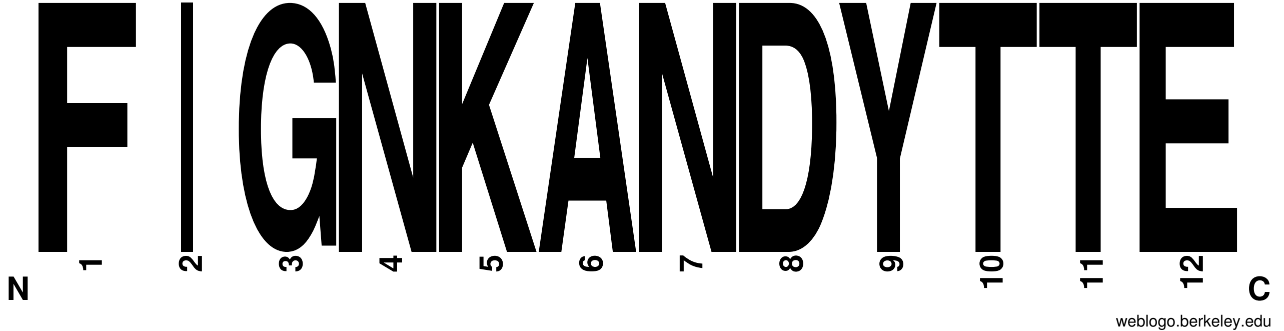

H2-12-IV

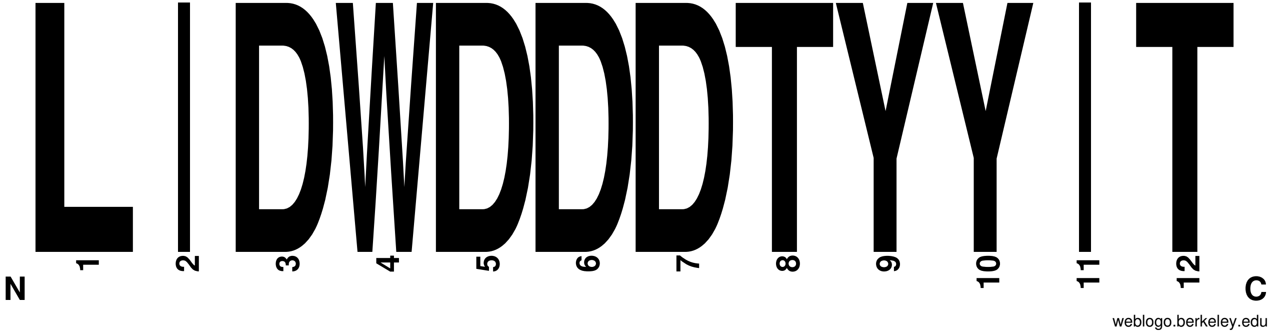

H2-15-I

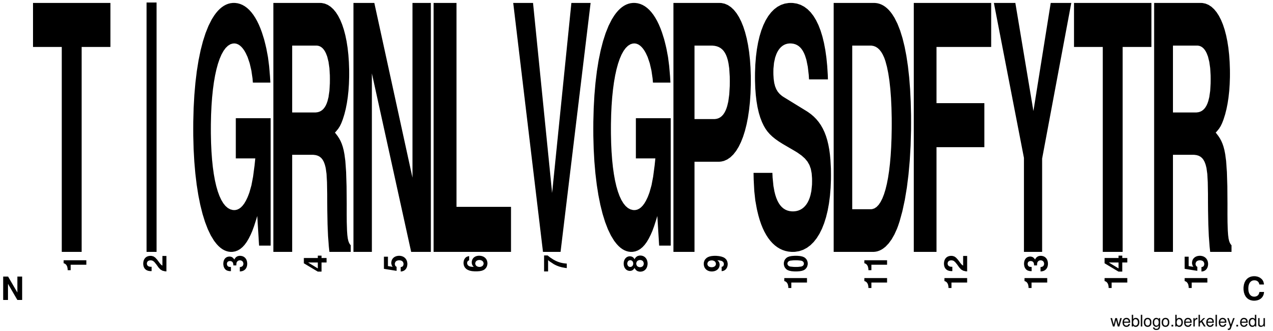
T I G R N L V G P S D F Y T R
1 2 3 4 5 6 7 8 9 10 11 12 13 14 15
N
C
weblogo.berkeley.edu

H2-15-II

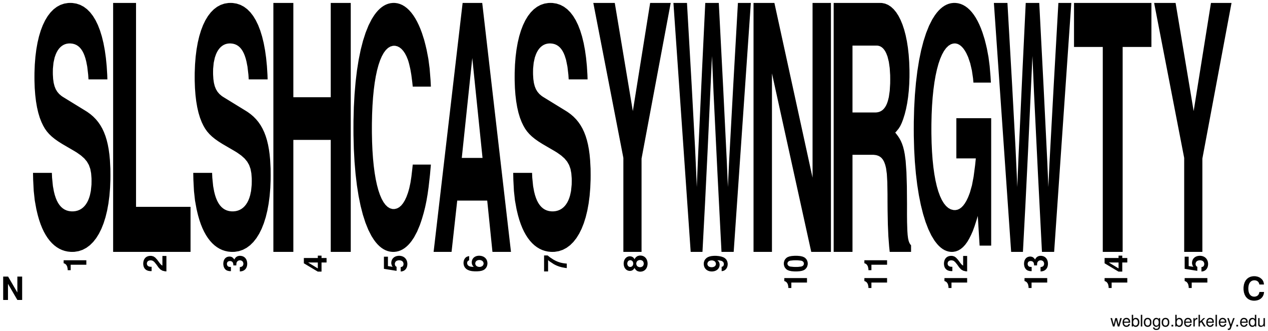
SLSHCASYWNRGWTY
1 2 3 4 5 6 7 8 9 10 11 12 13 14 15
N
C
weblogo.berkeley.edu

L1-7-I

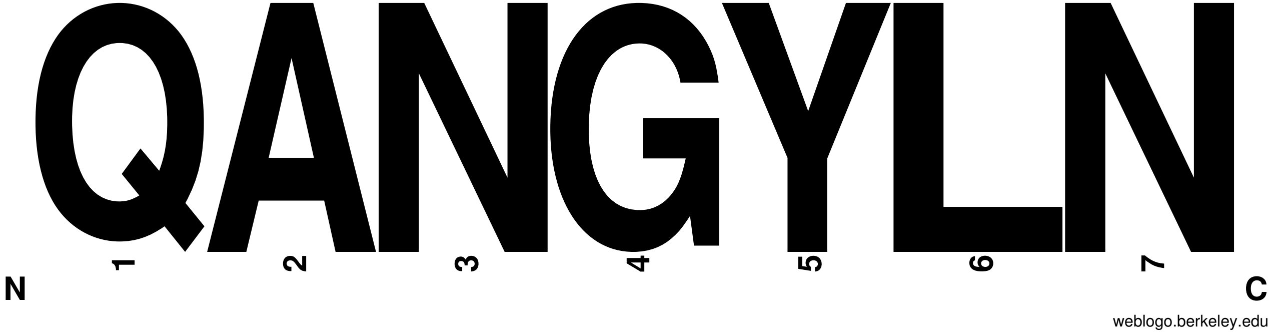

L1-9-I

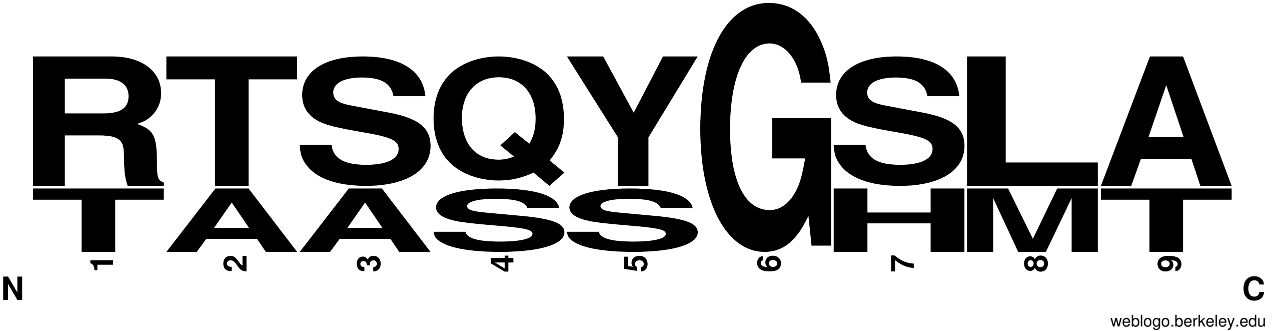

L1-9-II

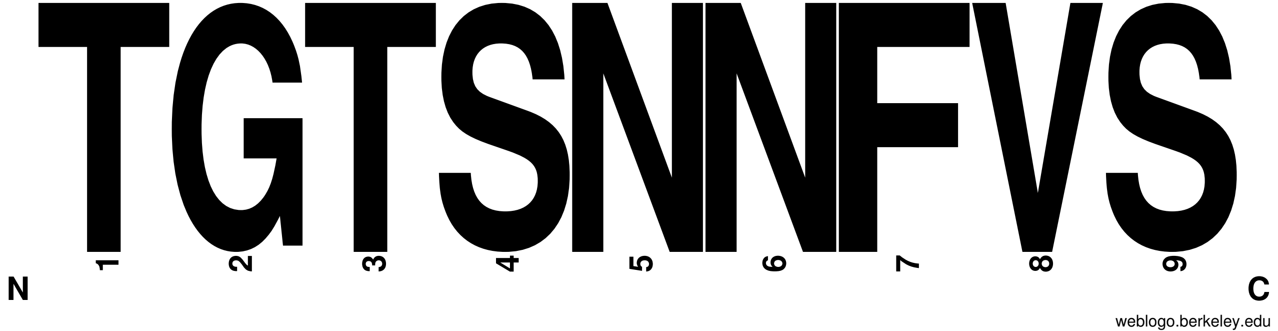

L1-10-I

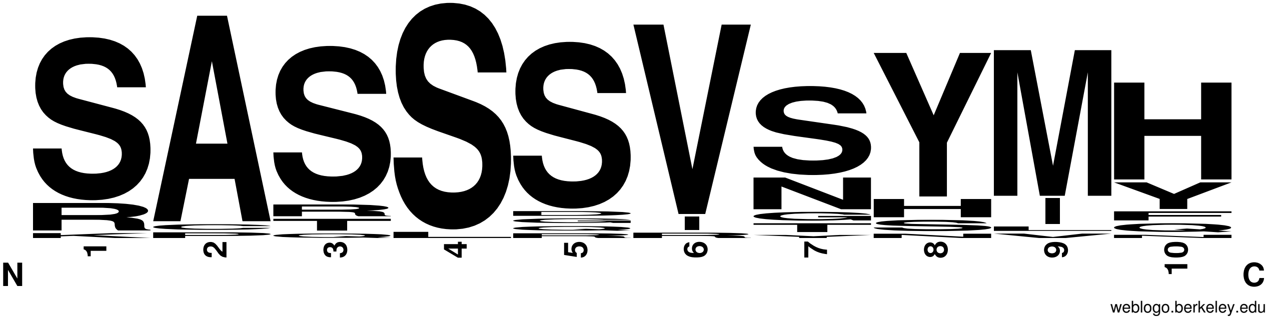

L1-11-I

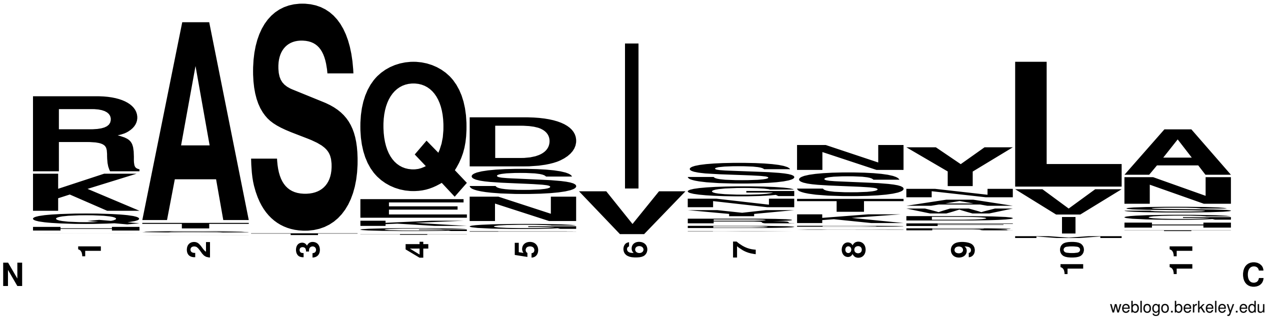

L1-11-II

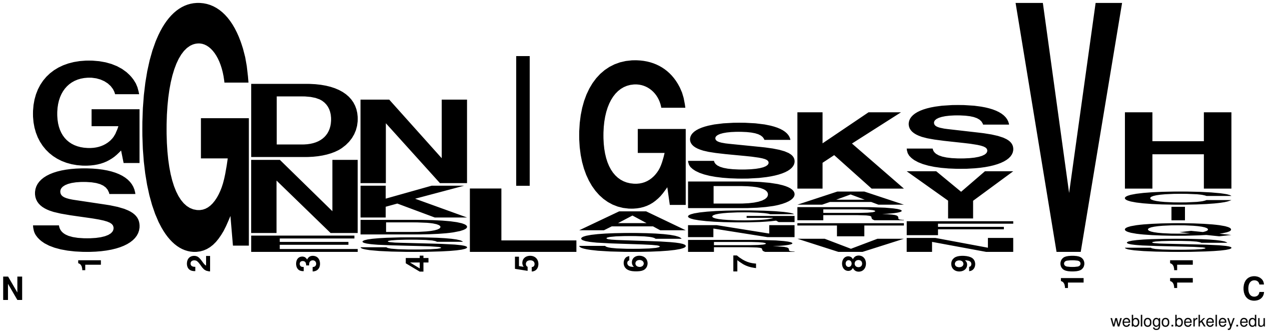

L1-11-III

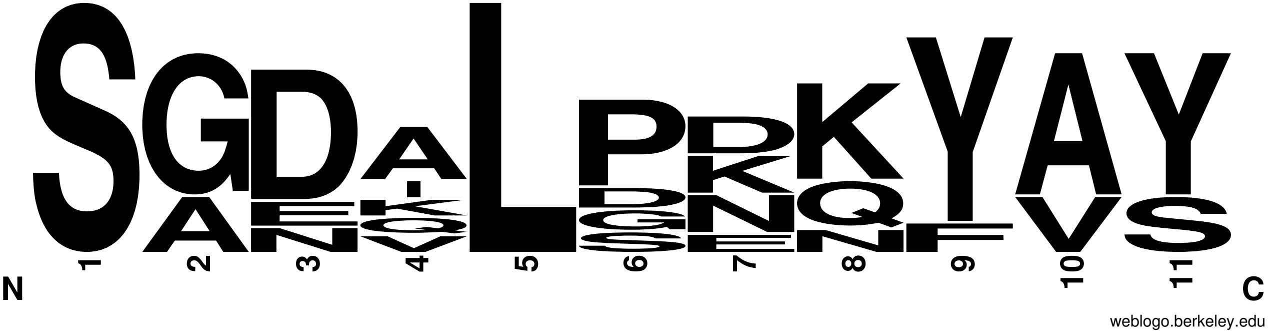

L1-11-IV

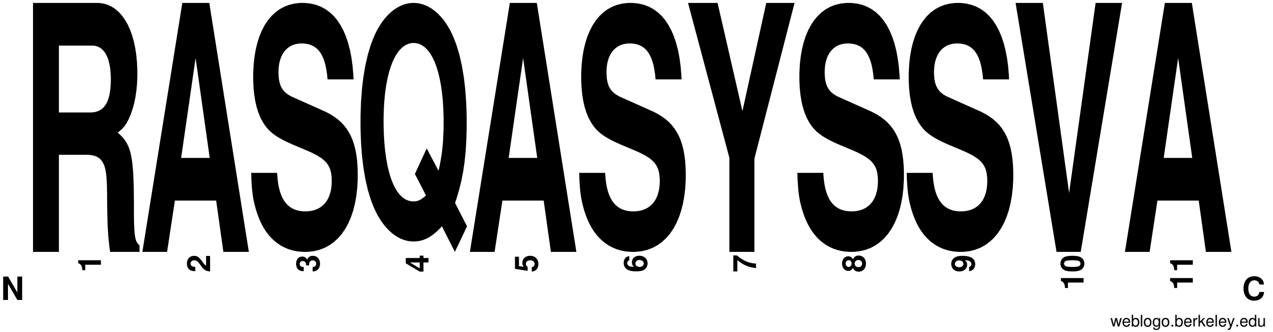

L1-12-I

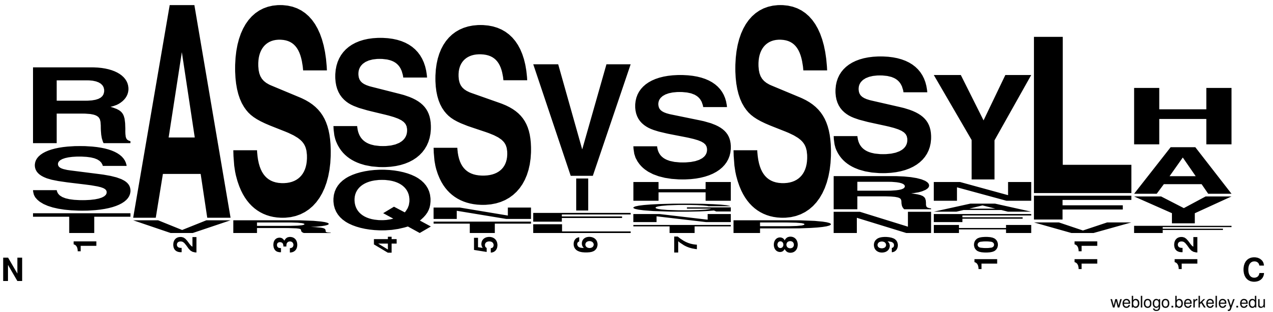

L1-12-II

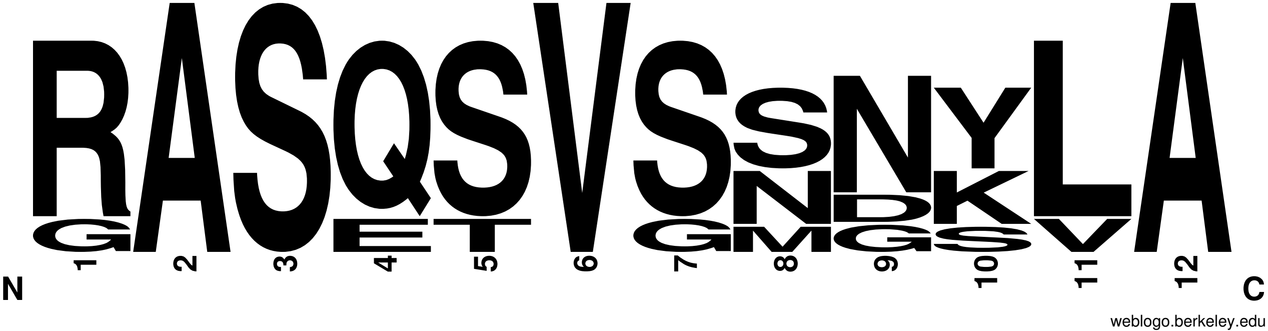

L1-12-III

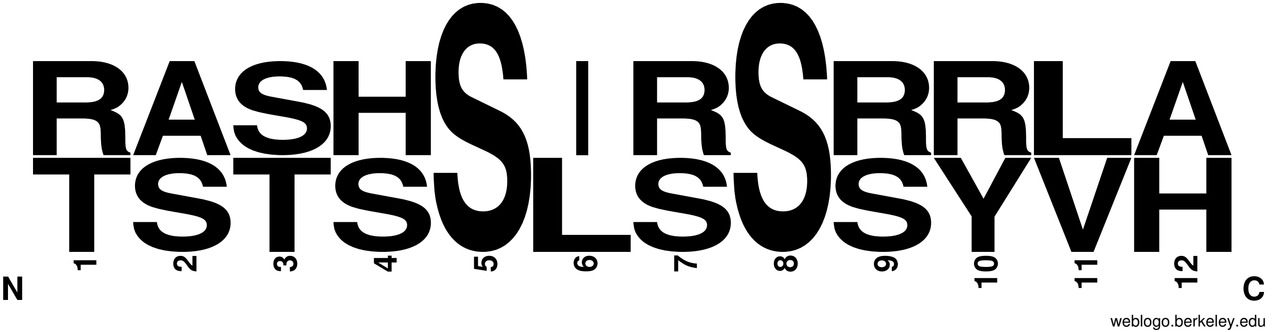
R A S H S I R S R R L A
T S T S S L S S S Y V H
1 2 3 4 5 6 7 8 9 10 11 12
N
C
weblogo.berkeley.edu

L1-12-IV

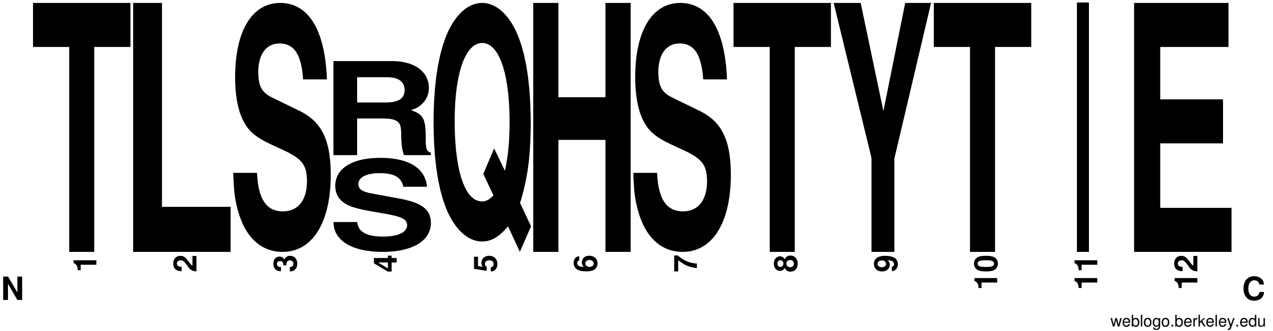

L1-13-I

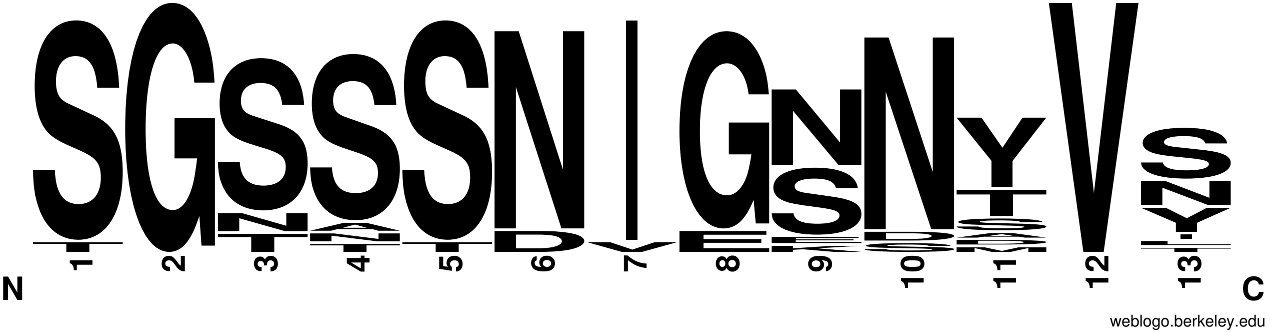

L1-13-II

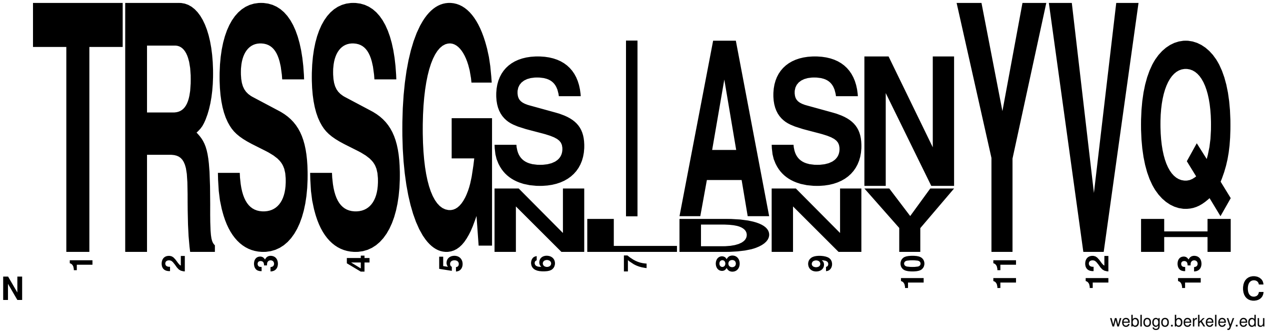

L1-13-III

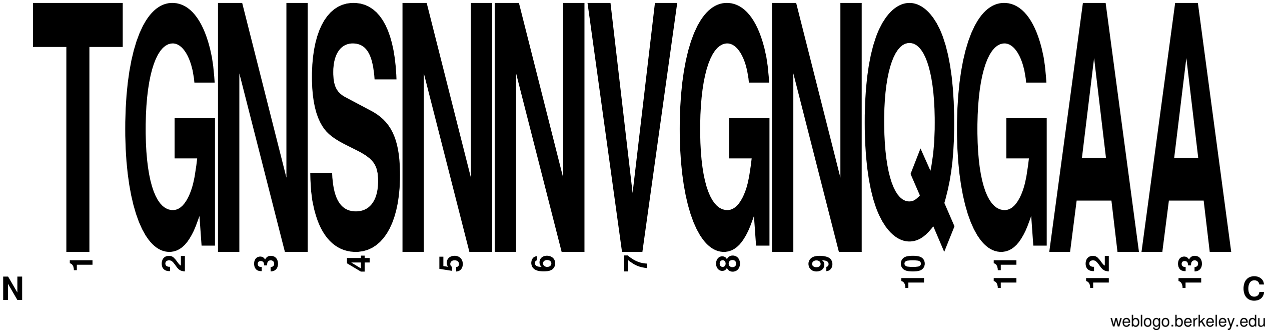

TGNSNNVGNQGAA
1
2
3
4
5
6
7
8
9
10
11
12
13
N
C
weblogo.berkeley.edu

L1-14-I

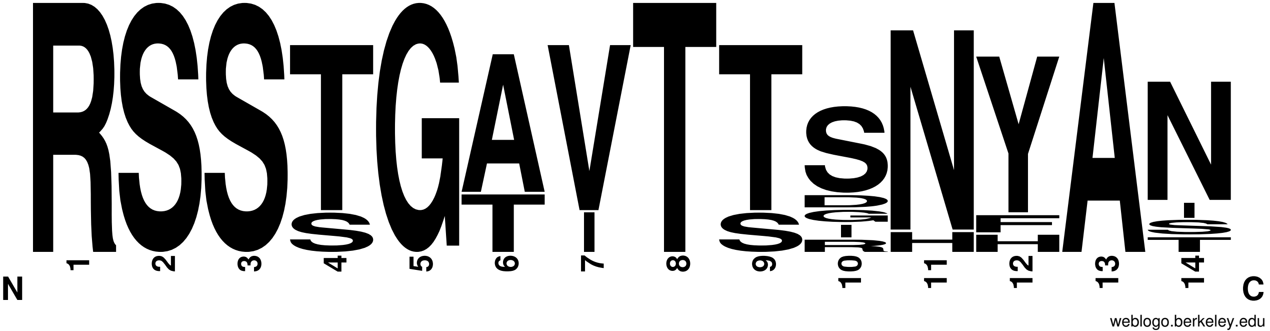

L1-14-II

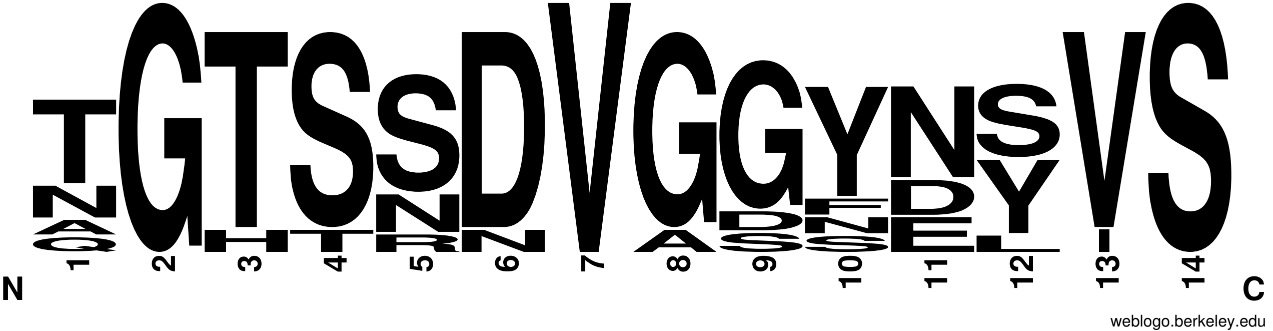

L1-14-III

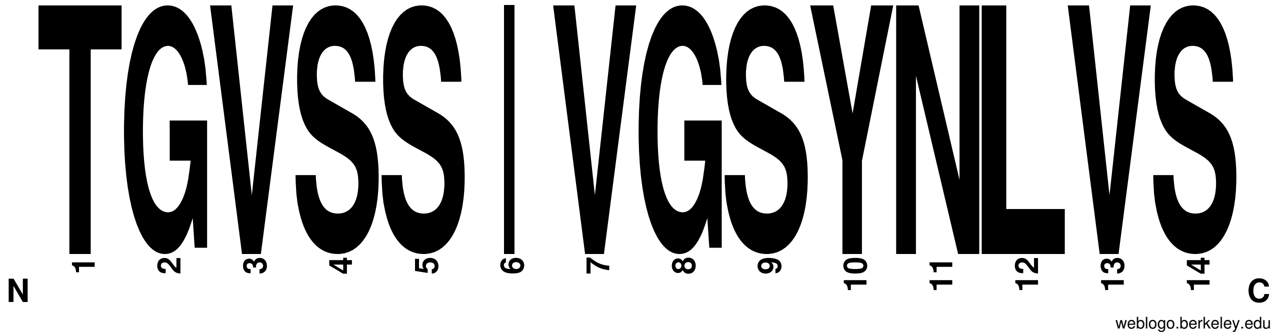
TGVSSIVGSYNLVS
N
1
2
3
4
5
6
7
8
9
10
11
12
13
14
C
weblogo.berkeley.edu

L1-14-IV

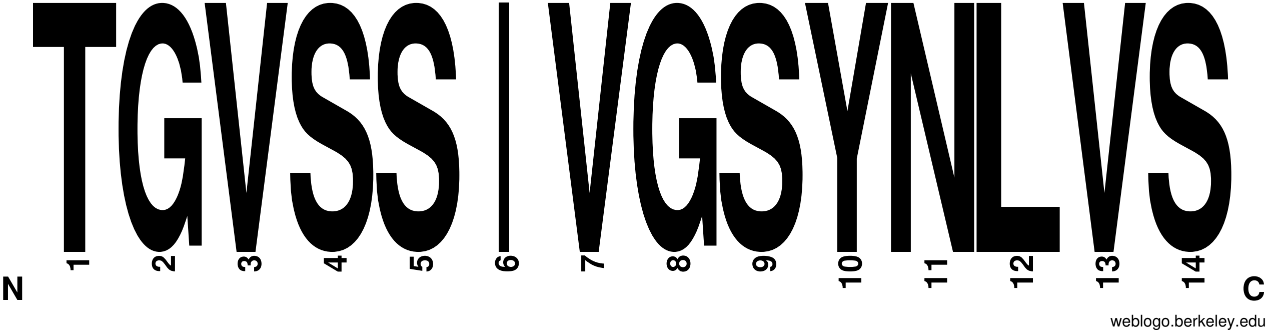

T G V S S I V G S Y N L V S
1 2 3 4 5 6 7 8 9 10 11 12 13 14
N
C
weblogo.berkeley.edu

L1-14-V

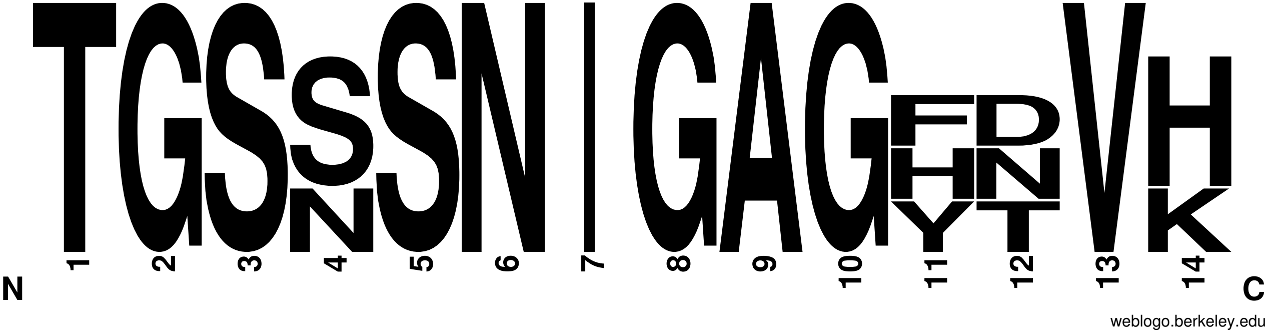

L1-14-VI

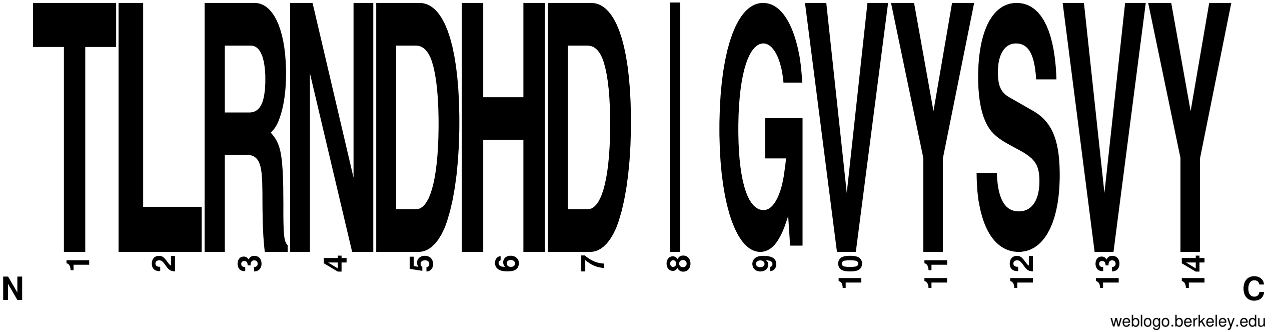
TLRNDHDIGVYSVY
1 2 3 4 5 6 7 8 9 10 11 12 13 14
N
C
weblogo.berkeley.edu

L1-14-VII

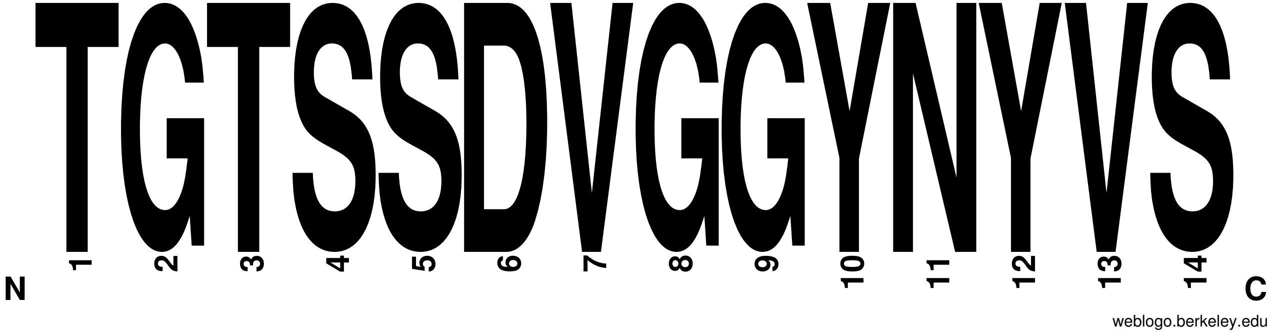
TGTSSDVGGYNYVS
1 2 3 4 5 6 7 8 9 10 11 12 13 14
N
C
weblogo.berkeley.edu

L1-15-I

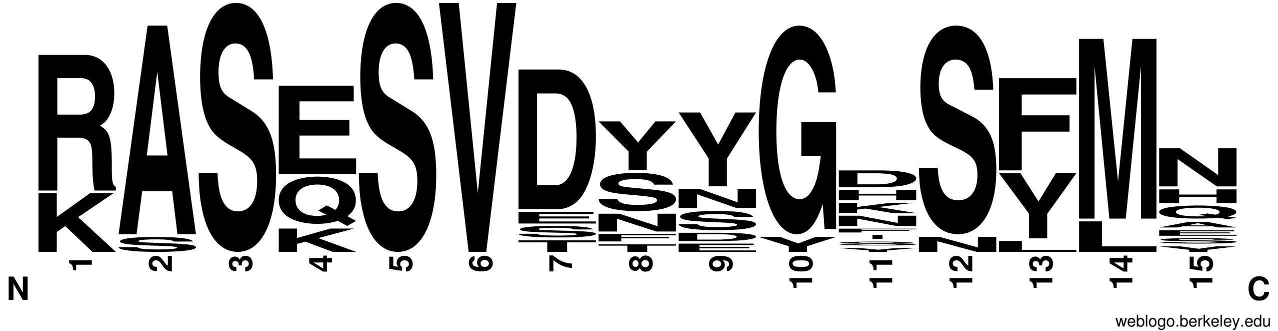

L1-15-II

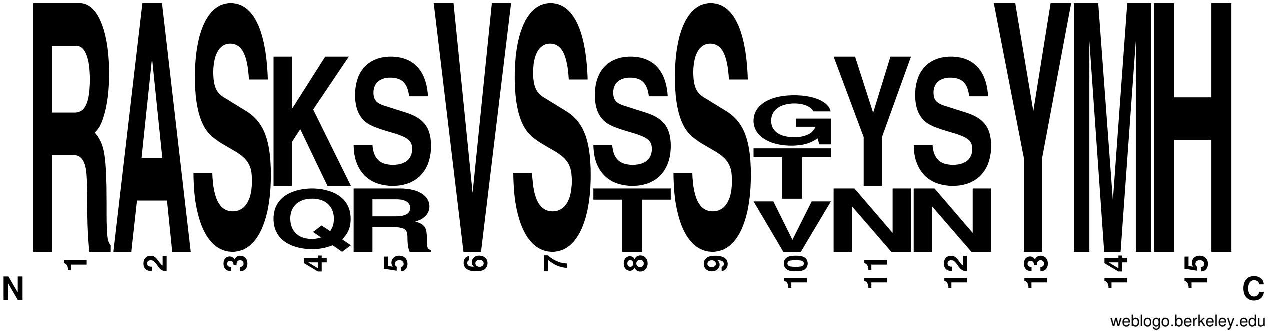

L1-16-I

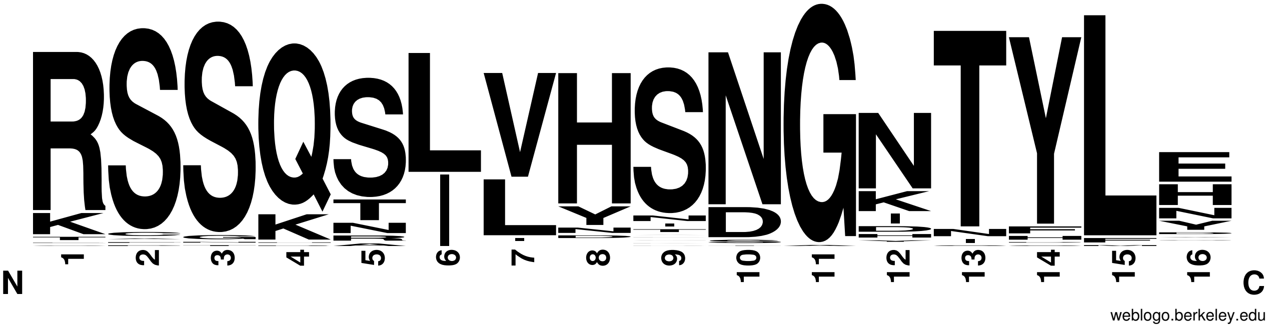

L1-16-II

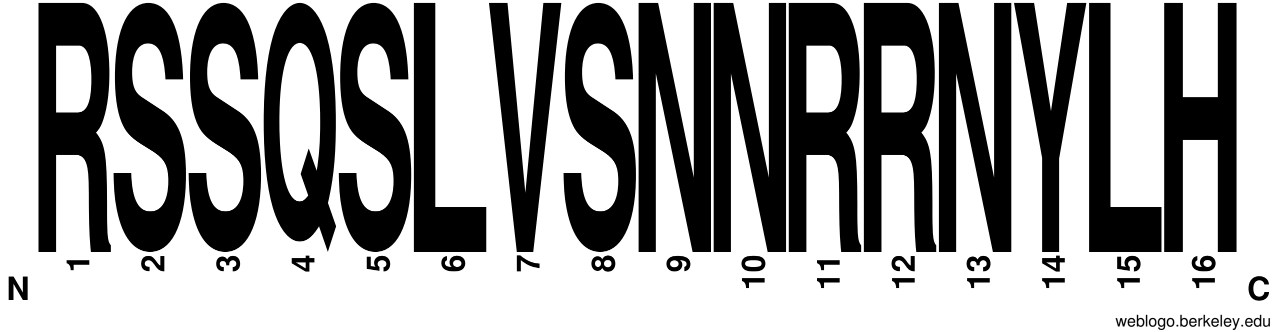
RSSQSLVSNNRRNYLH
N
1 2 3 4 5 6 7 8 9 10 11 12 13 14 15 16
C
weblogo.berkeley.edu

L1-16-III

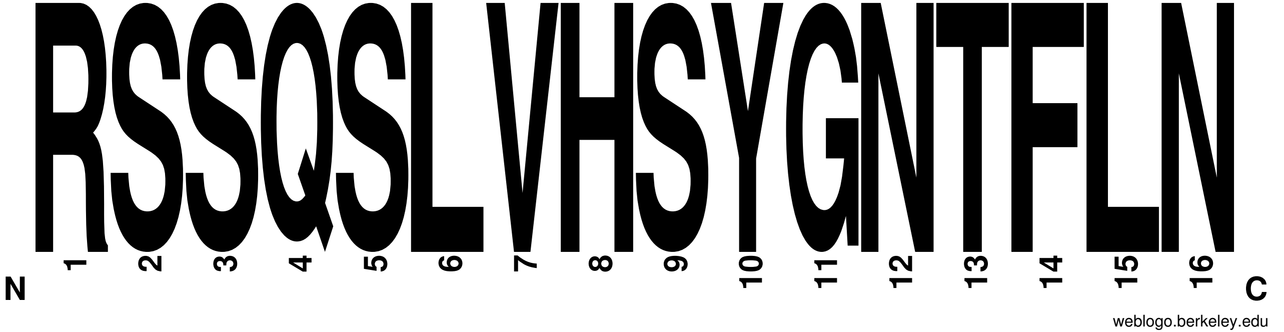

RSSQSLVHSYGNTFLN
1 2 3 4 5 6 7 8 9 10 11 12 13 14 15 16
N
C
weblogo.berkeley.edu

L1-16-IV

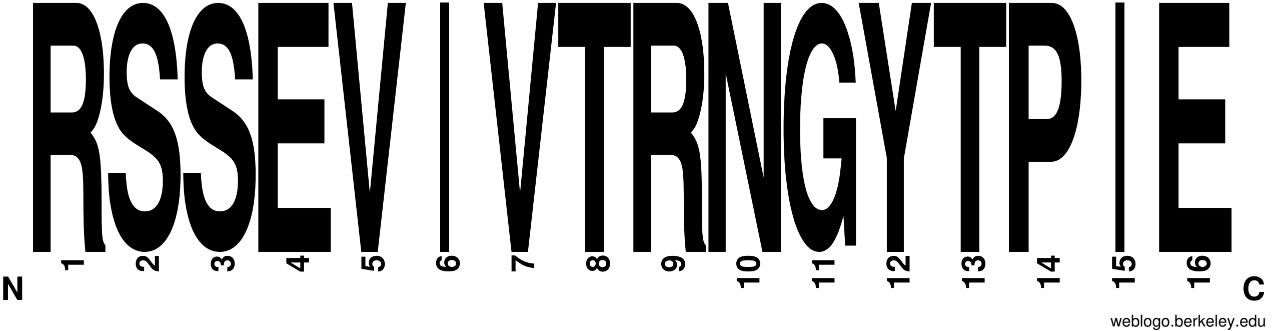
RSSEVIVTRNGYTPIE
N
1 2 3 4 5 6 7 8 9 10 11 12 13 14 15 16
C
weblogo.berkeley.edu

L1-16-V

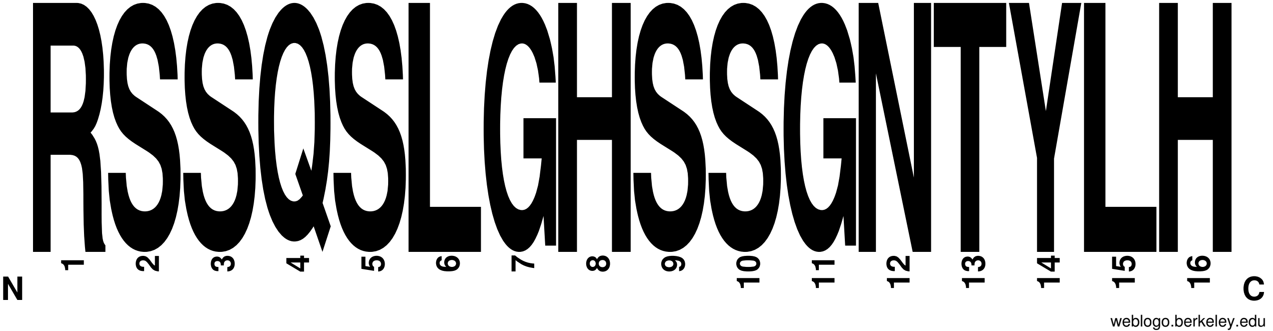
RSSQSLGHSSGNTYLH
1 2 3 4 5 6 7 8 9 10 11 12 13 14 15 16
N
C
weblogo.berkeley.edu

L1-17-I

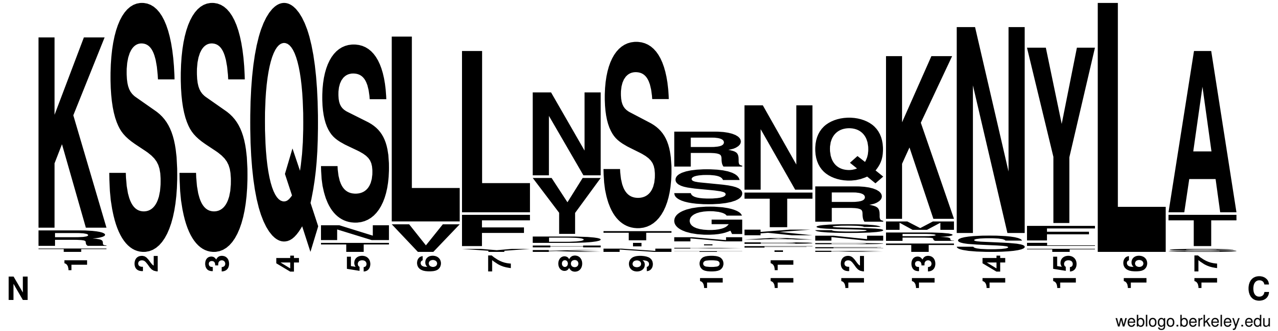

L2-7-I

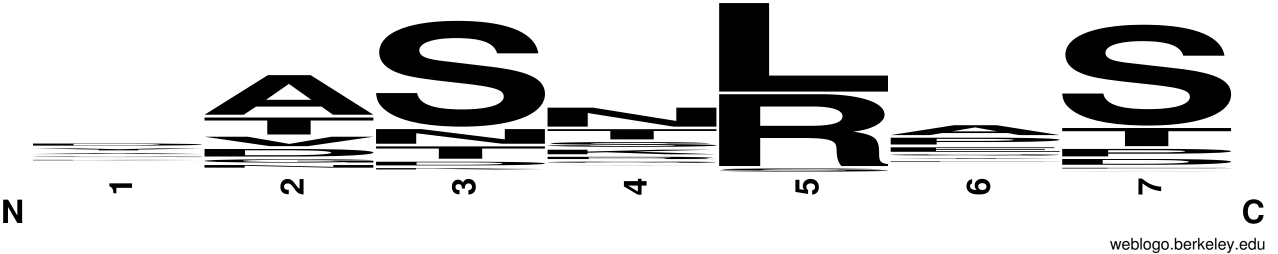

L2-7-II

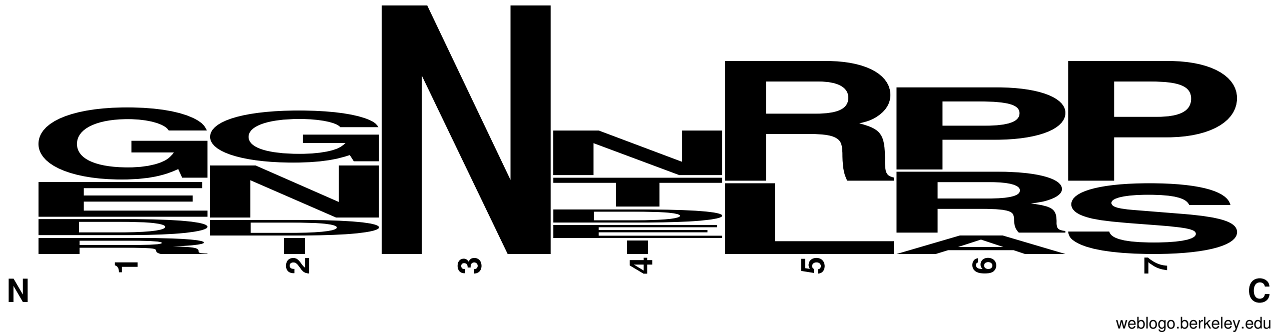

L2-7-III

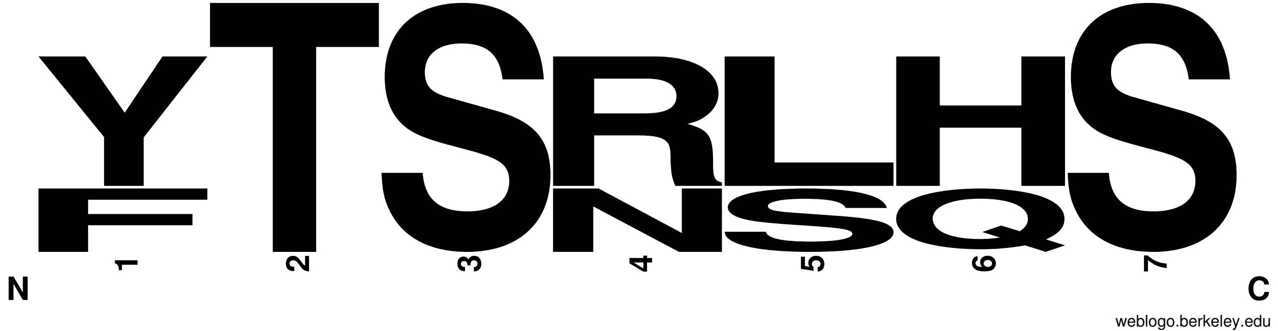

L2-11-I

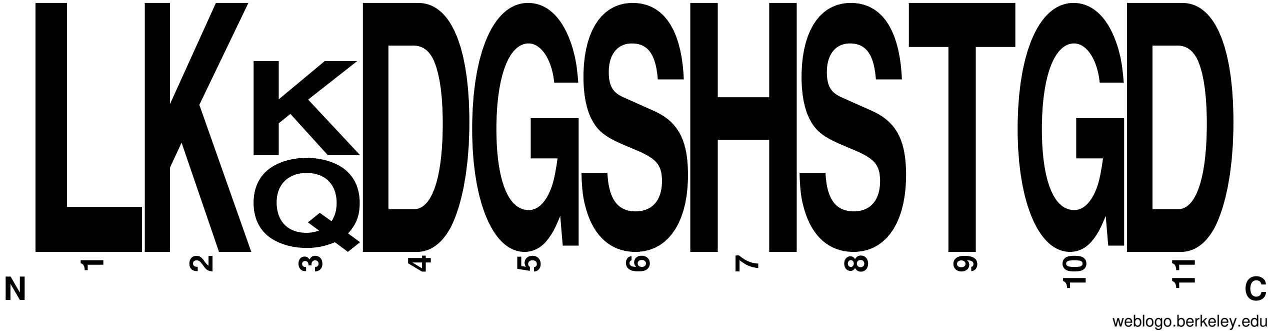

L2-11-II

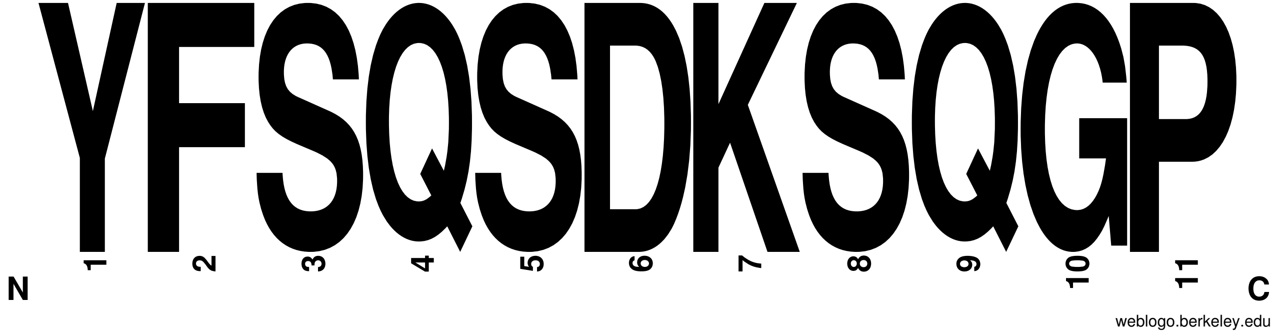

L3-5-I

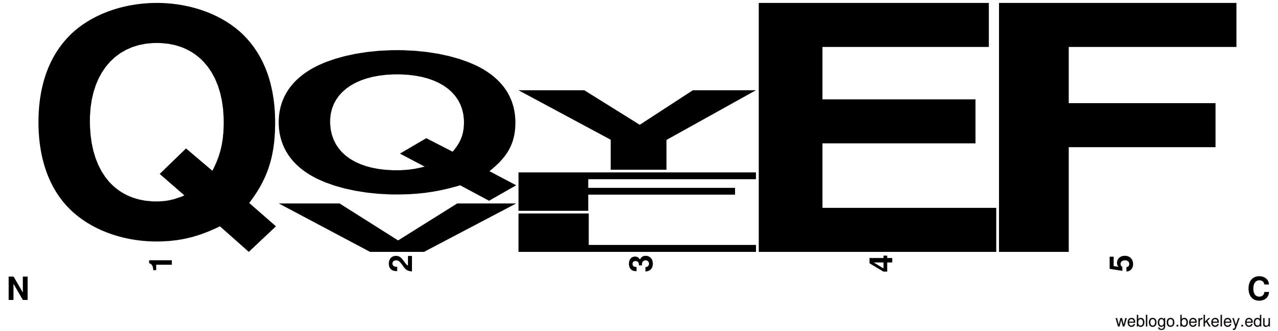

L3-7-I

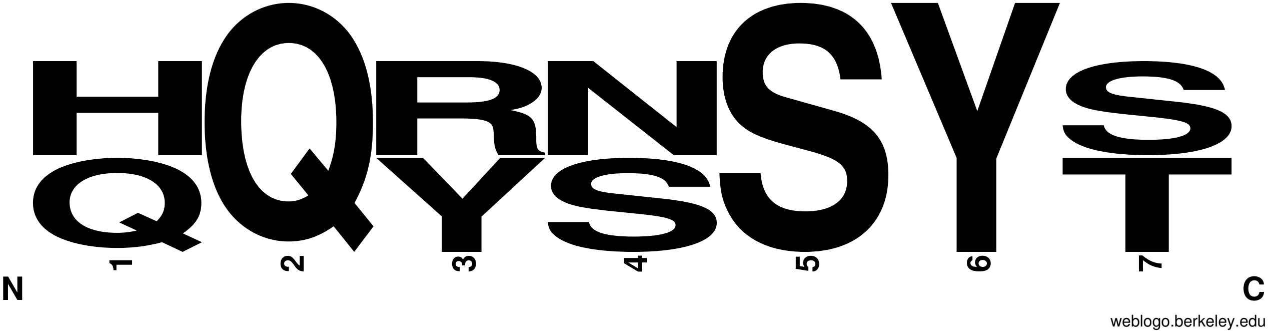

L3-8-I

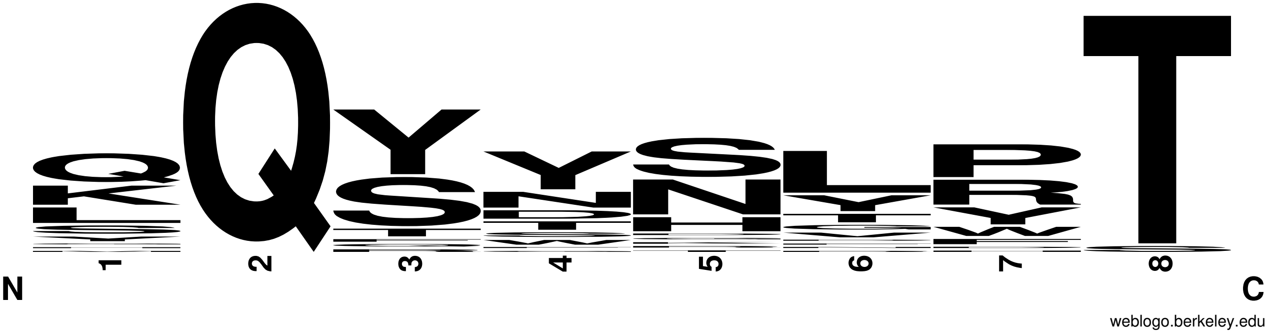

L3-8-II

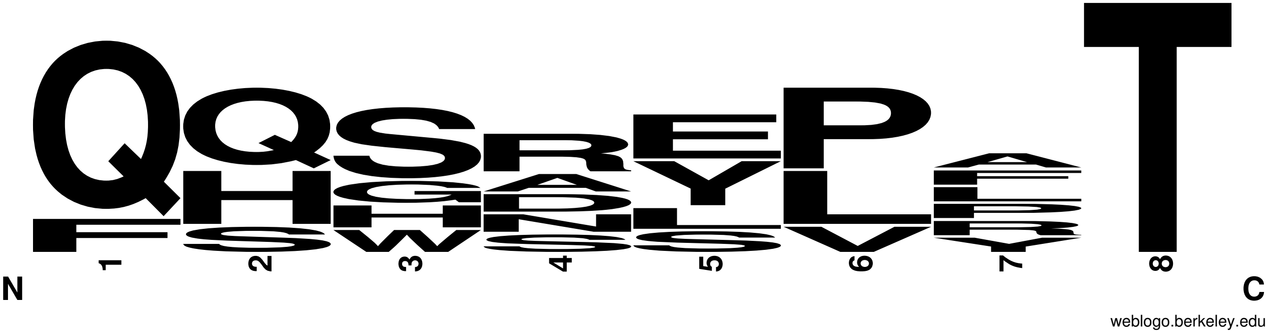

L3-8-III

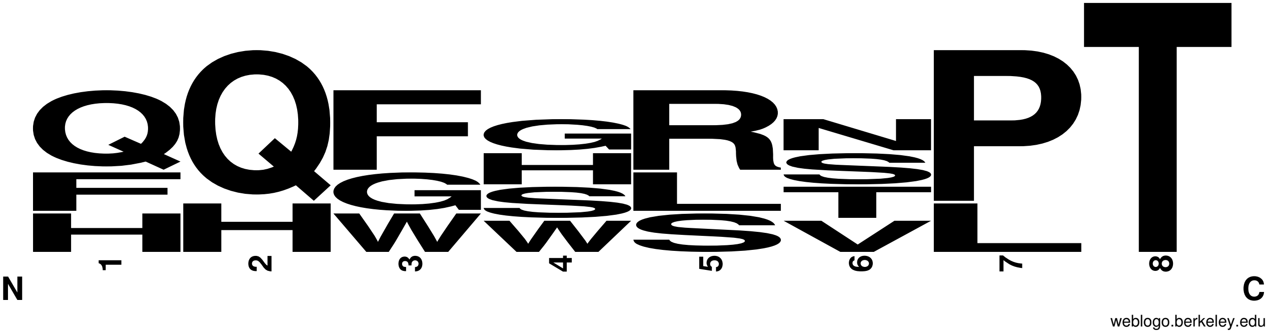

L3-8-IV

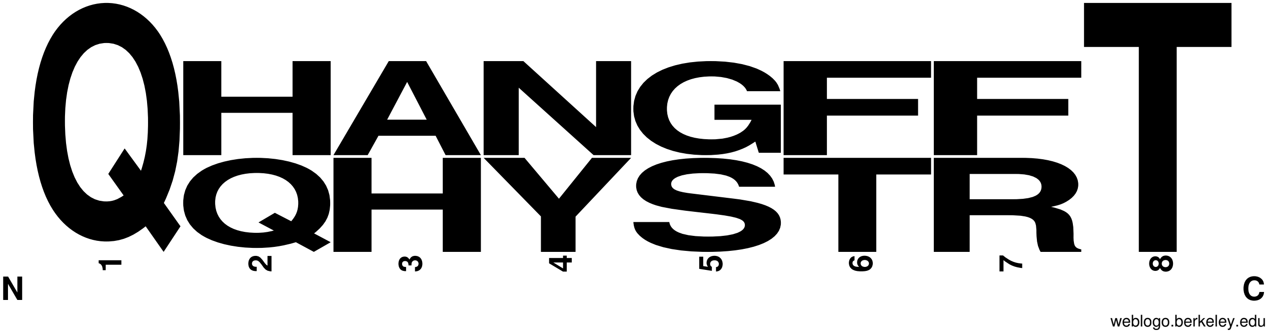

L3-8-V

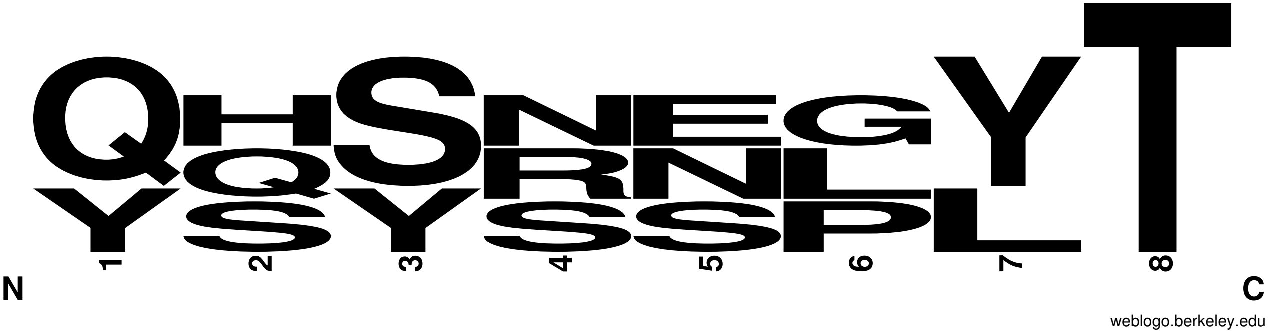

L3-8-VI

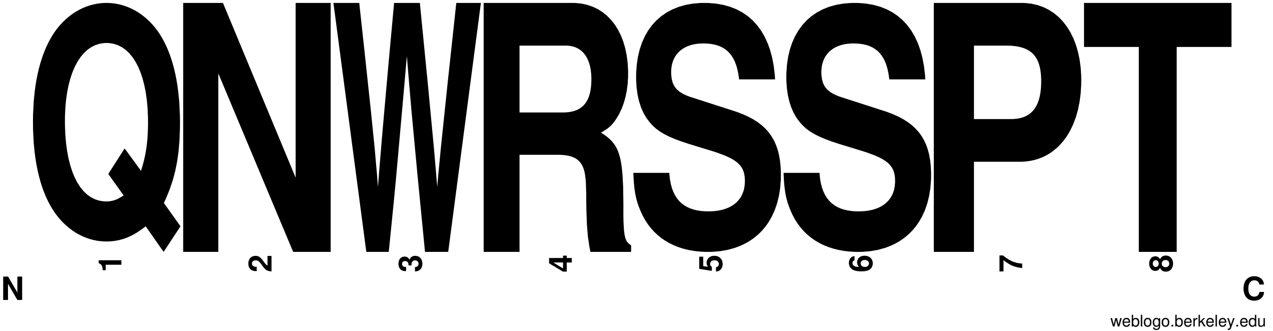

L3-9-I

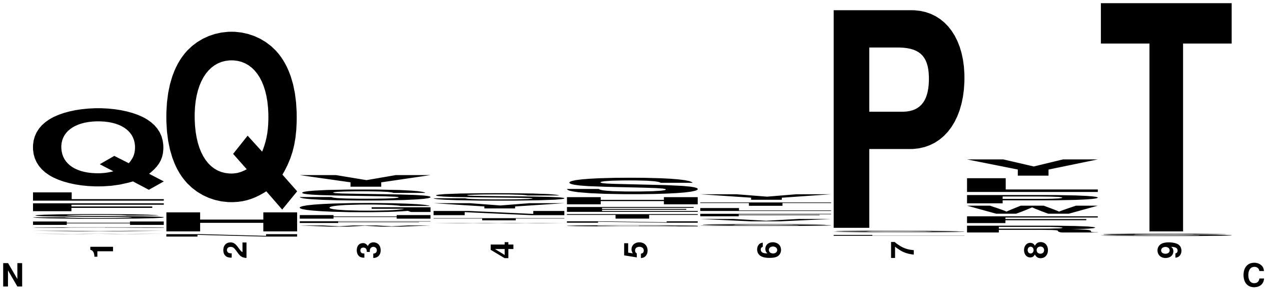

weblogo.berkeley.edu

L3-9-II

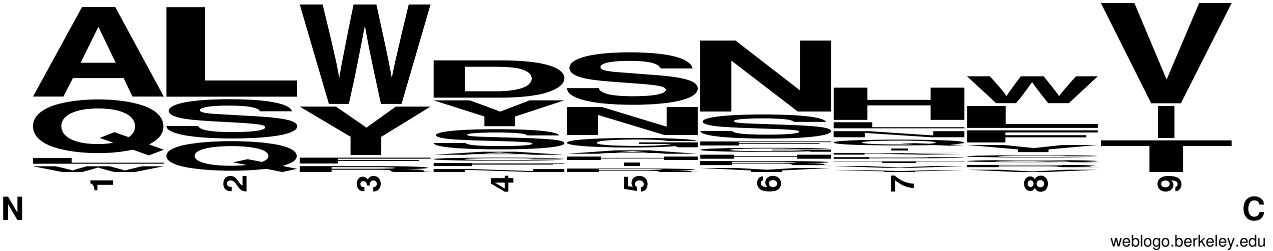

L3-9-III

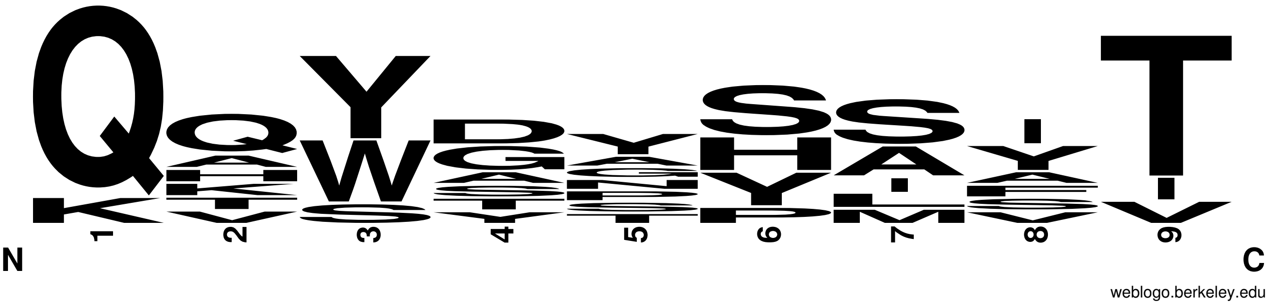

L3-9-IV

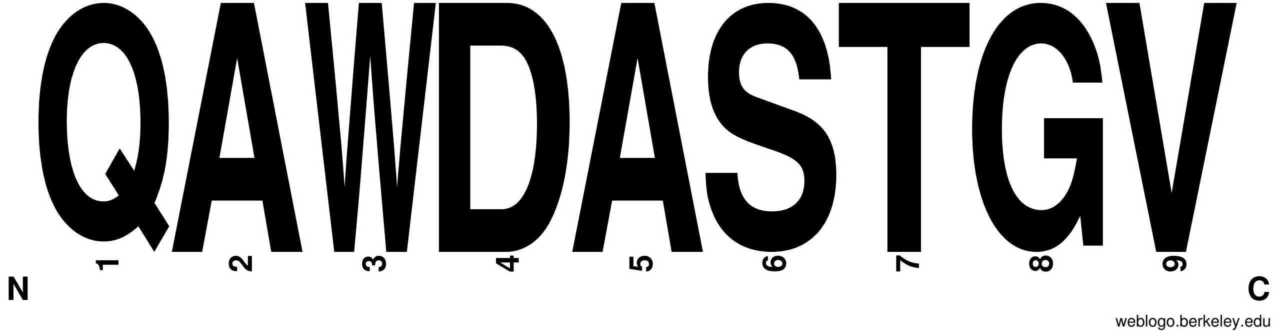

L3-9-V

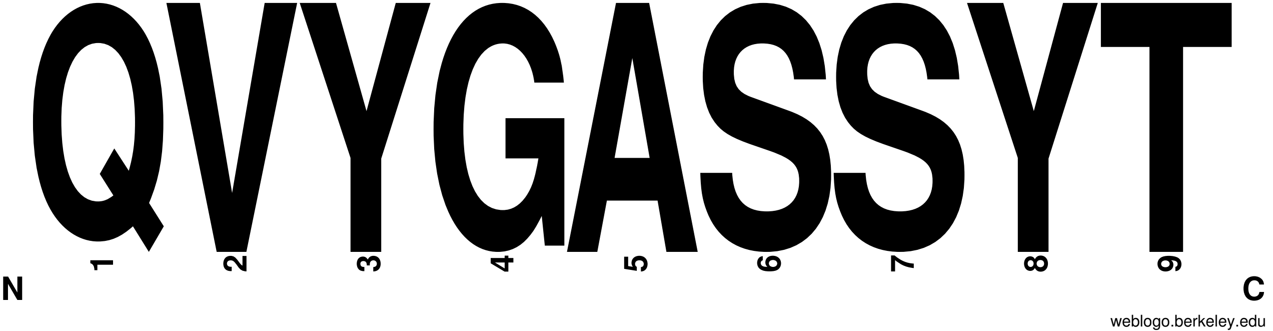

L3-9-VI

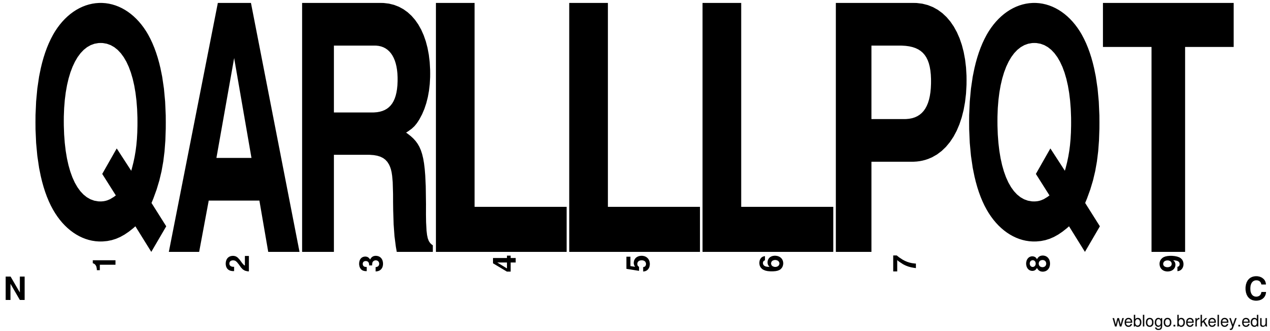

L3-10-I

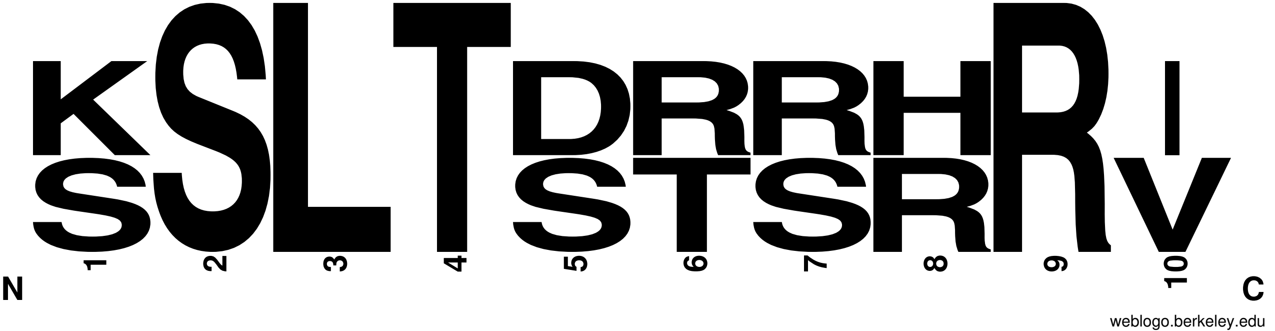

L3-10-II

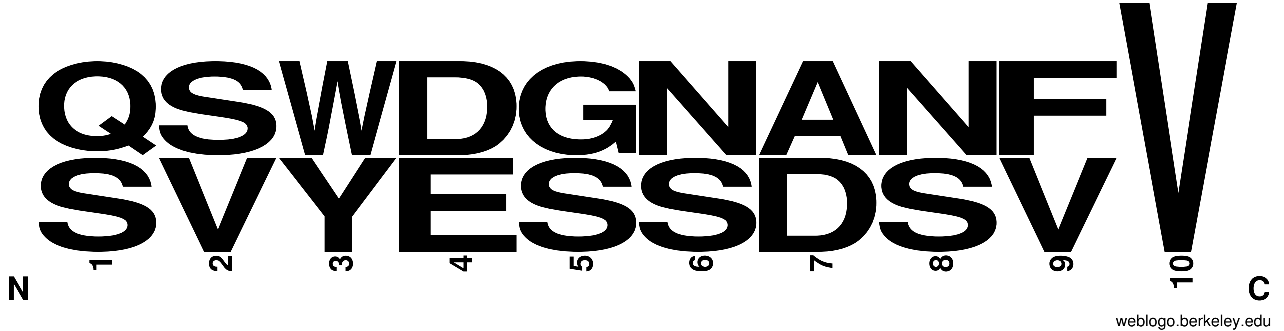
QSWDGNANFV
SVYESSSDSV
1 2 3 4 5 6 7 8 9 10
N
C
weblogo.berkeley.edu

L3-10-III

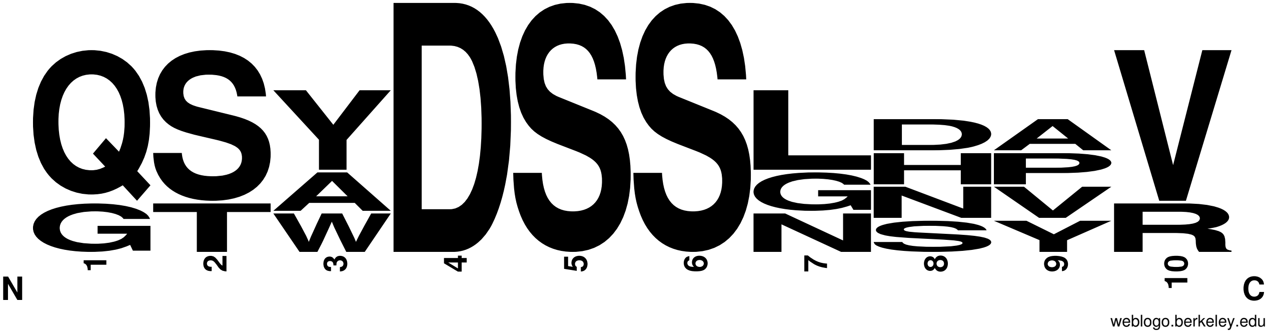

L3-10-IV

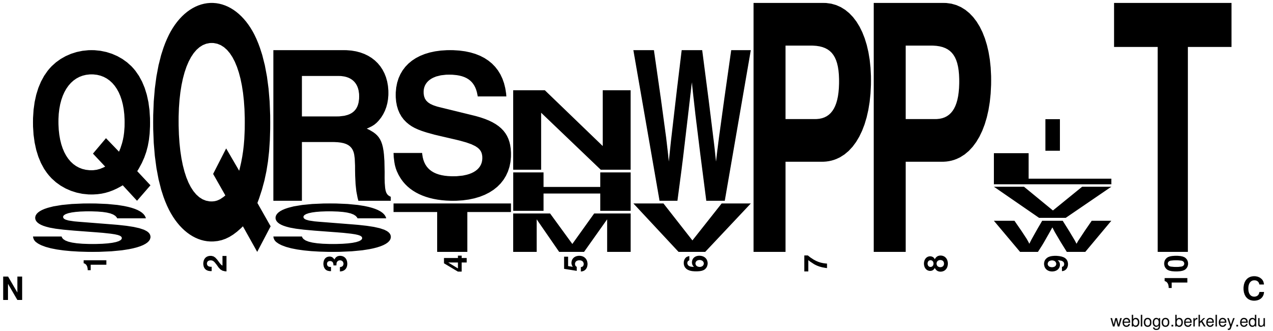

L3-10-V

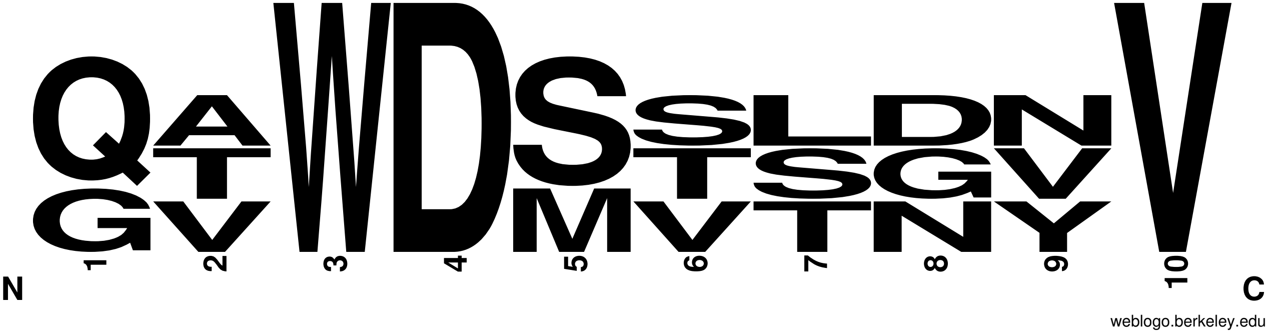

L3-10-VI

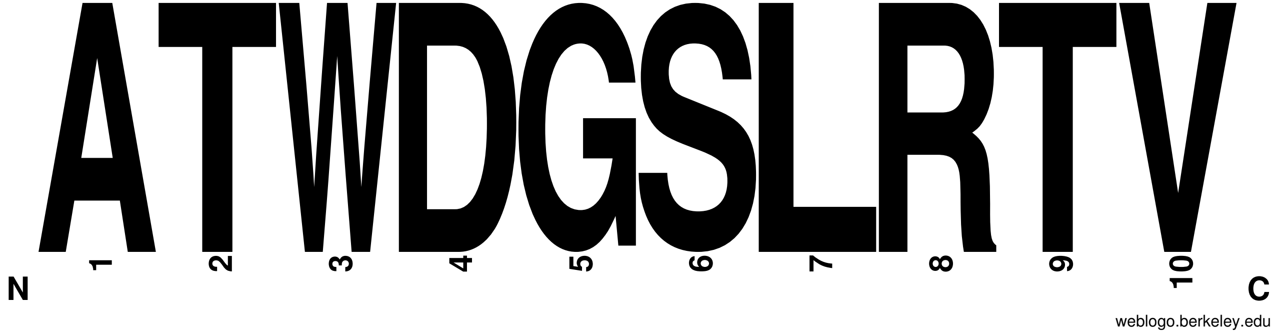

L3-10-VII

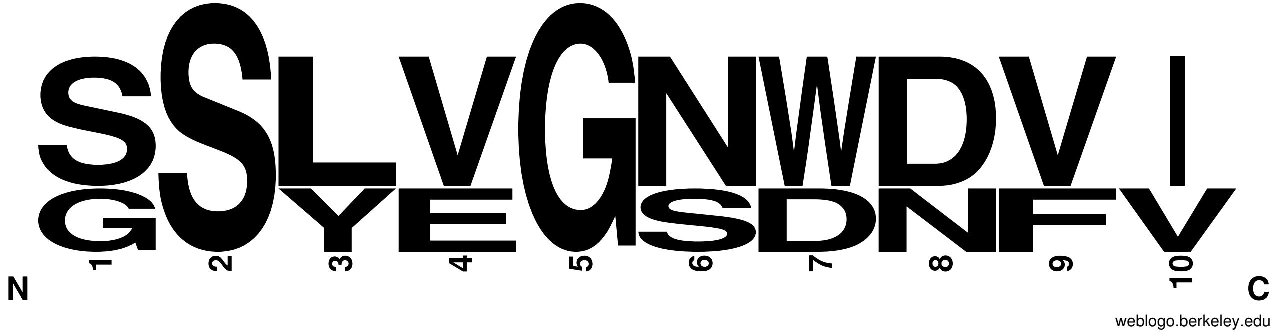

L3-10-VIII

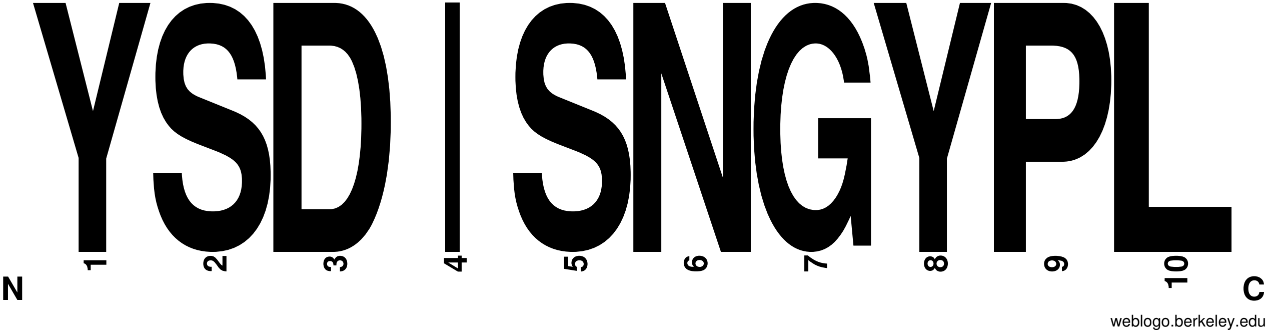

L3-10-IX

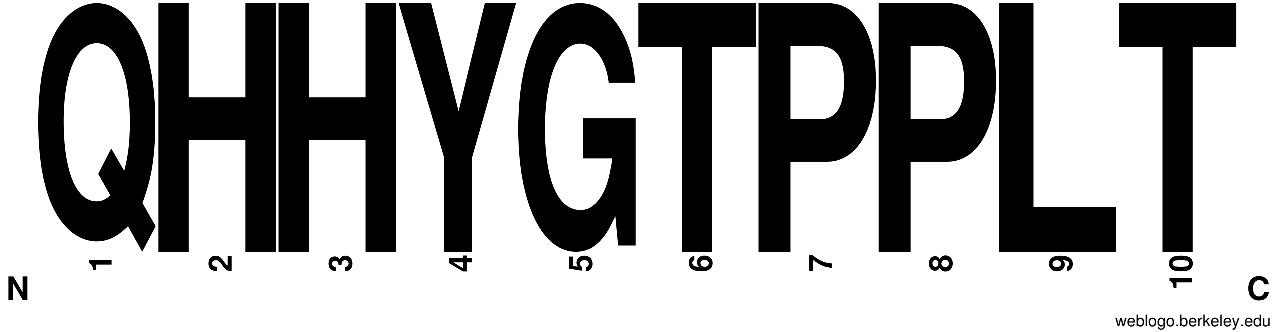

L3-10-X

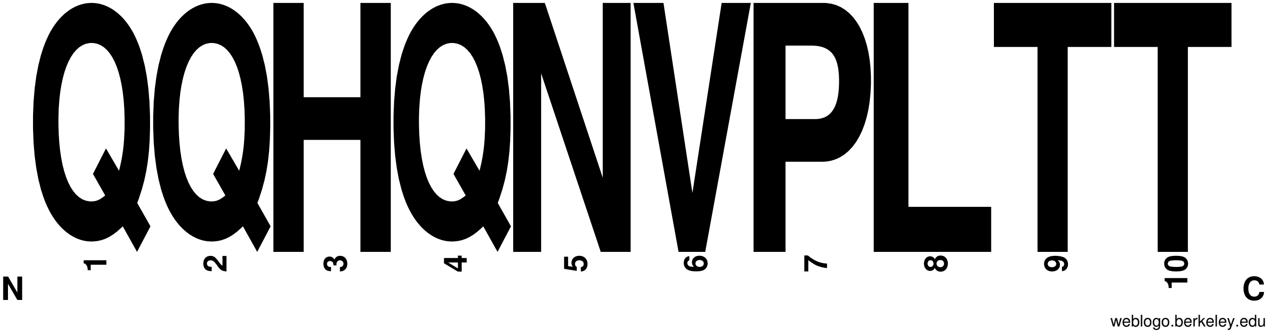

L3-10-XI

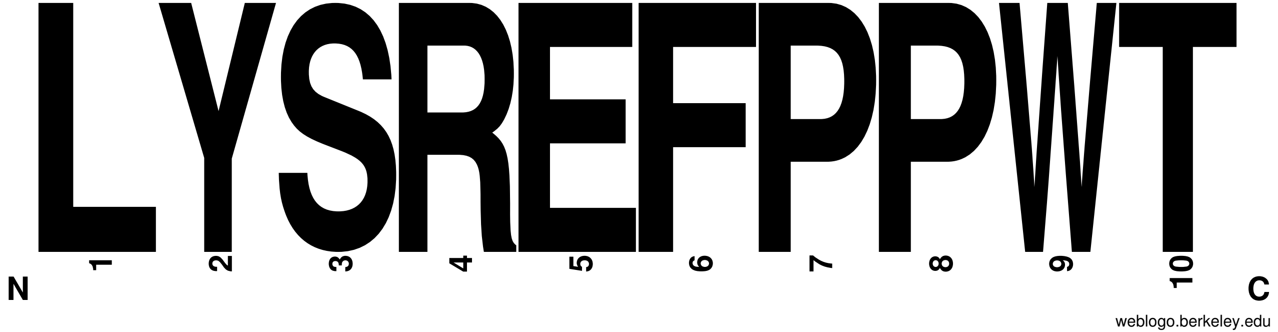

L3-10-XII

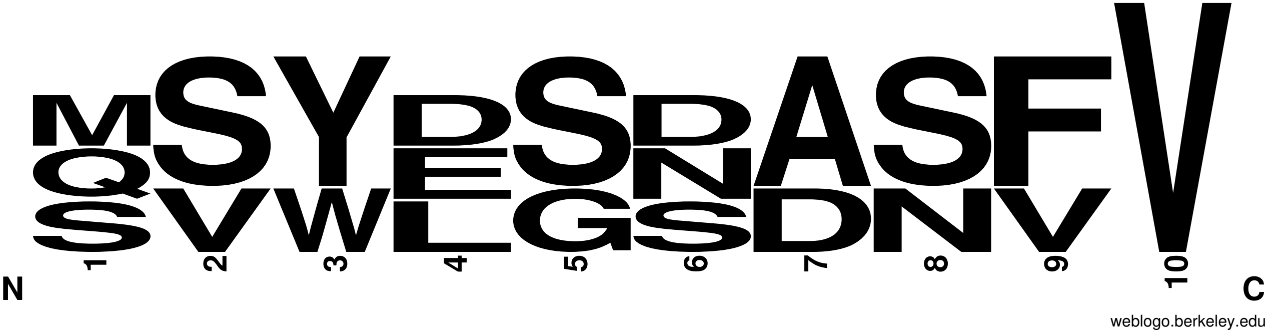

L3-11-I

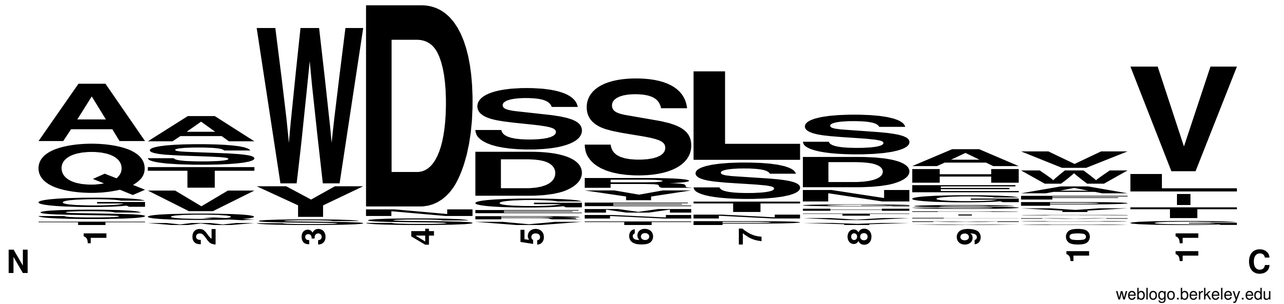

L3-11-II

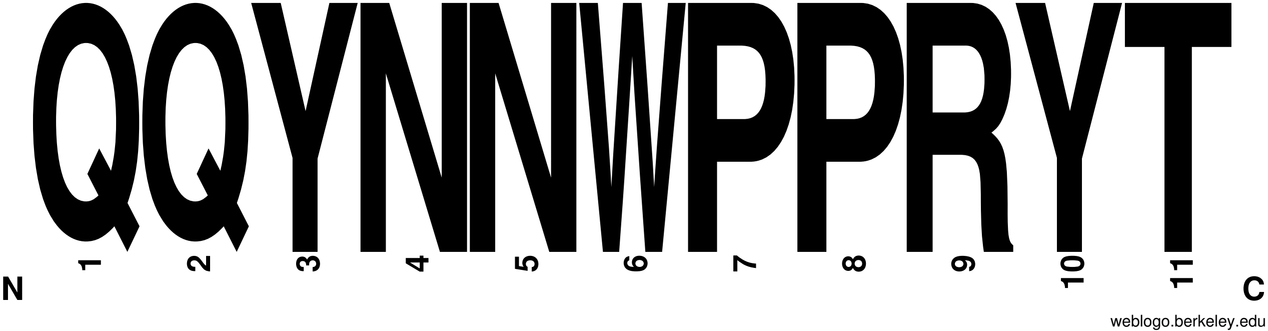

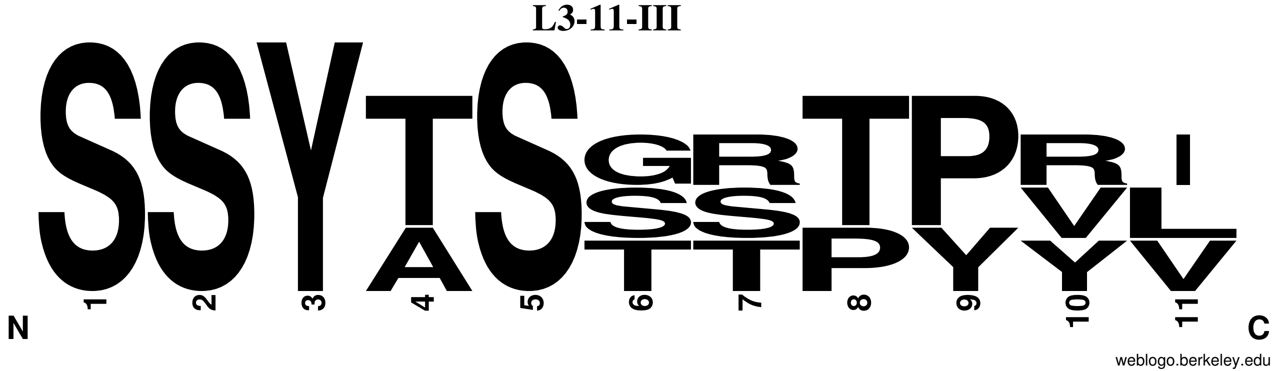
L3-11-III
N
1
2
3
4
5
6
7
8
9
10
11
C
weblogo.berkeley.edu

L3-11-IV

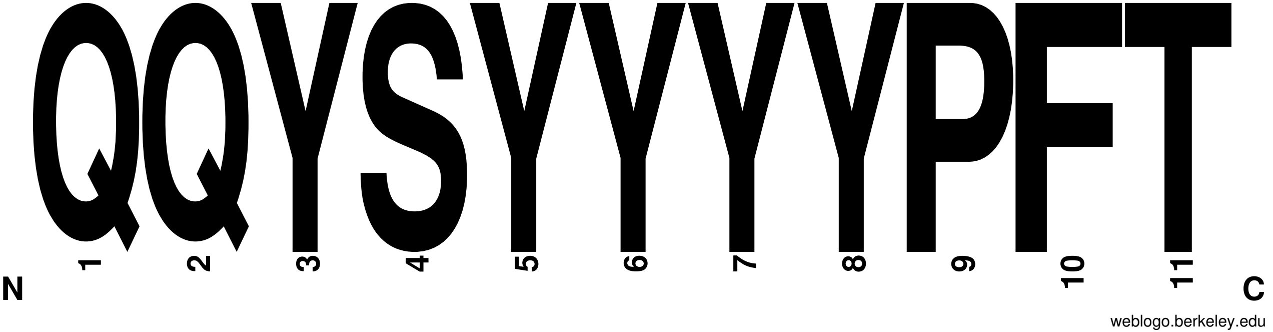

L3-11-V

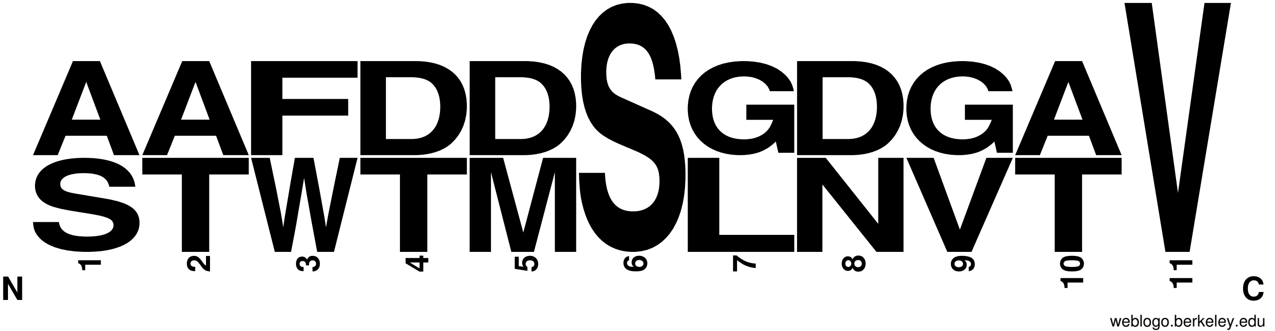

L3-11-VI

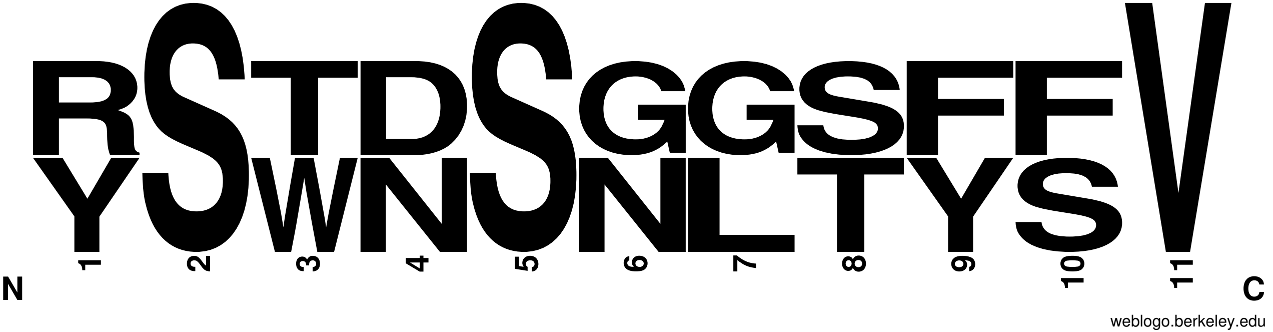

L3-11-VII

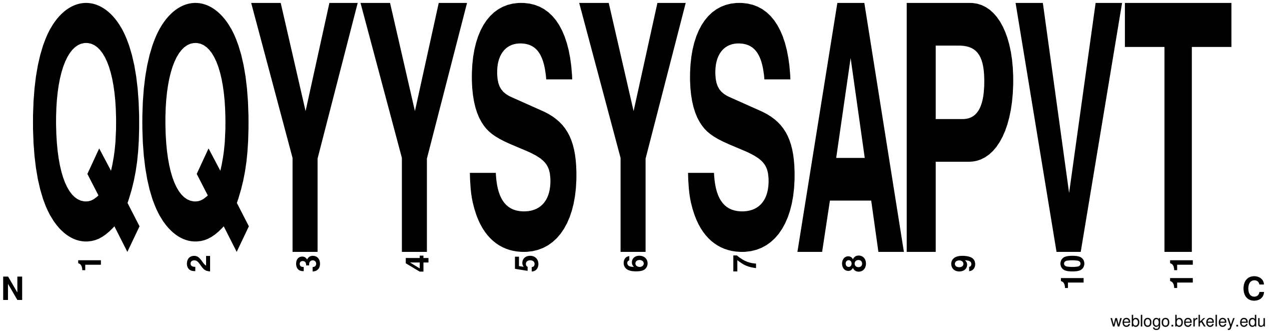

L3-11-VIII

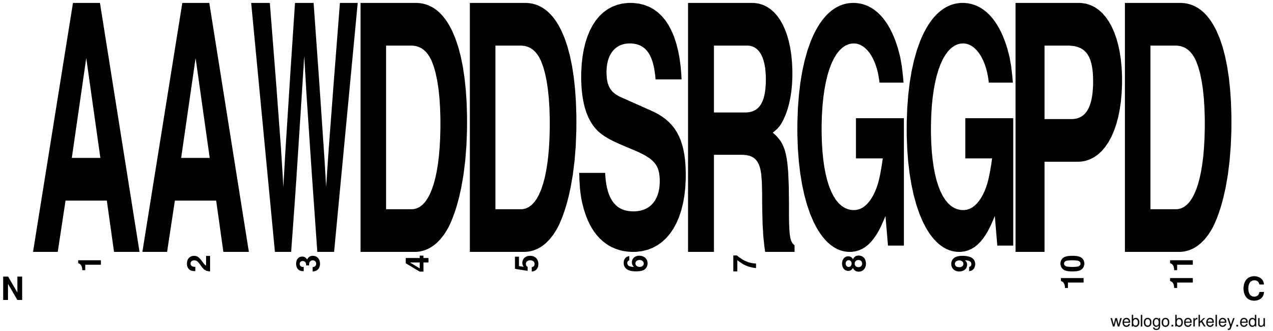

L3-11-IX

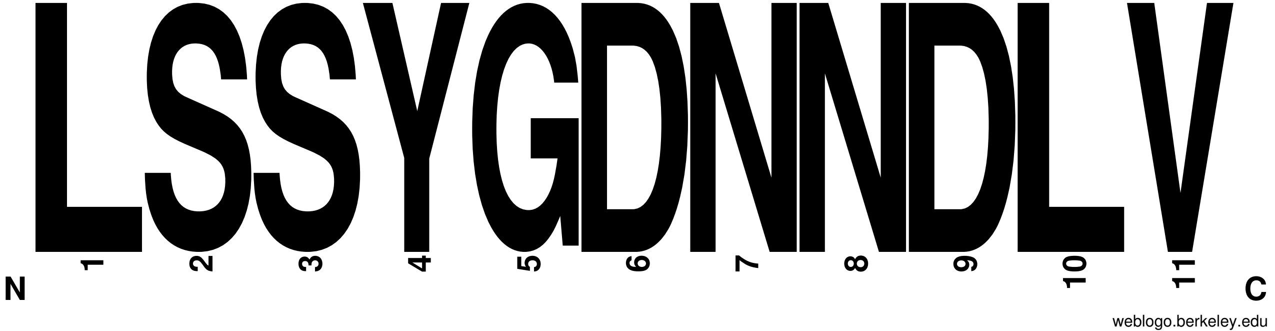

L3-12-I

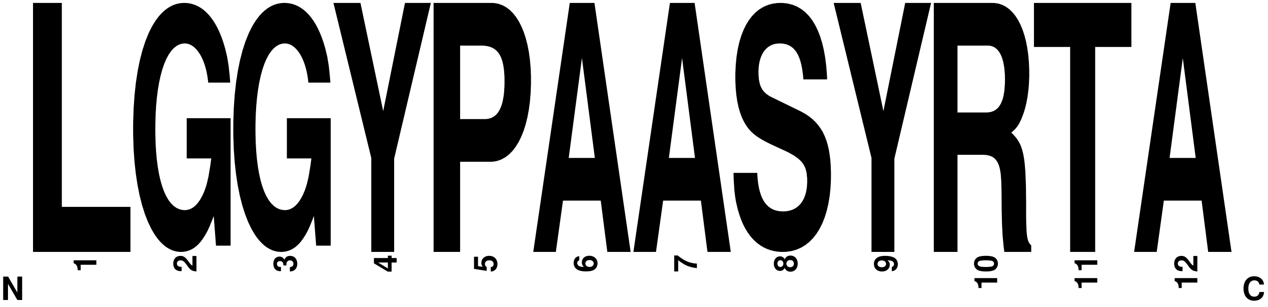

weblogo.berkeley.edu

L3-12-II

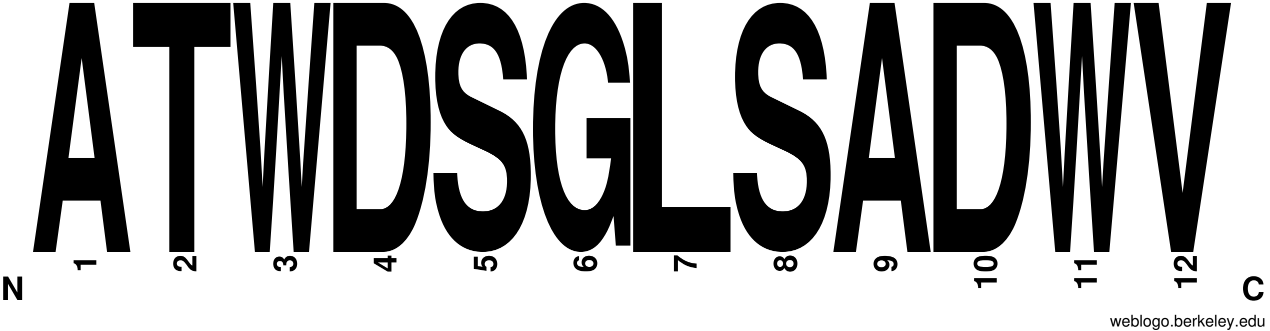

L3-12-III

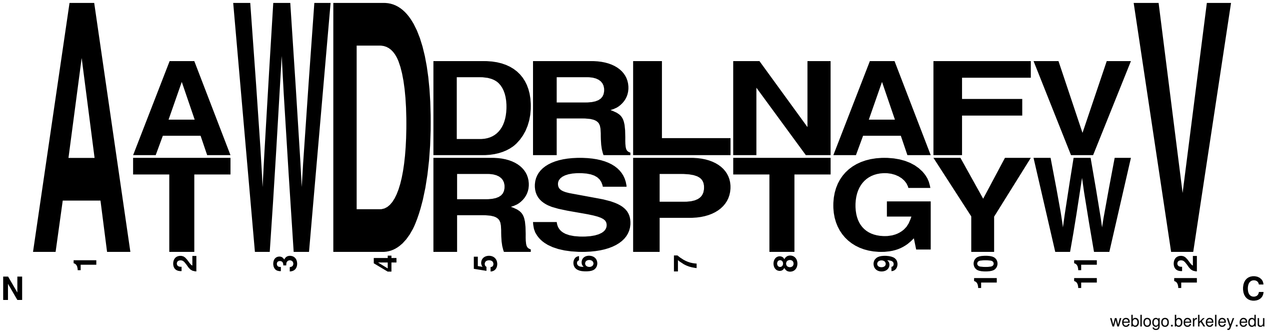

L3-12-IV

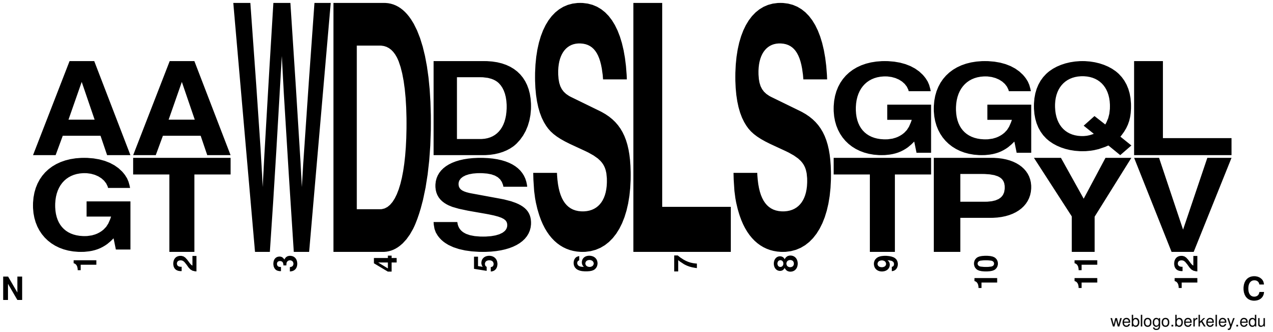

L3-13-I

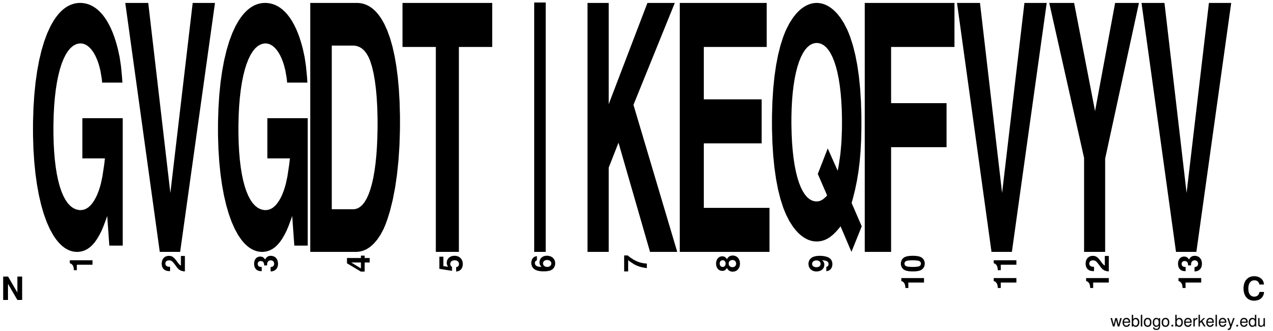
GVGDTIKEQFVYV
1 2 3 4 5 6 7 8 9 10 11 12 13
N
C
weblogo.berkeley.edu

L3-13-II

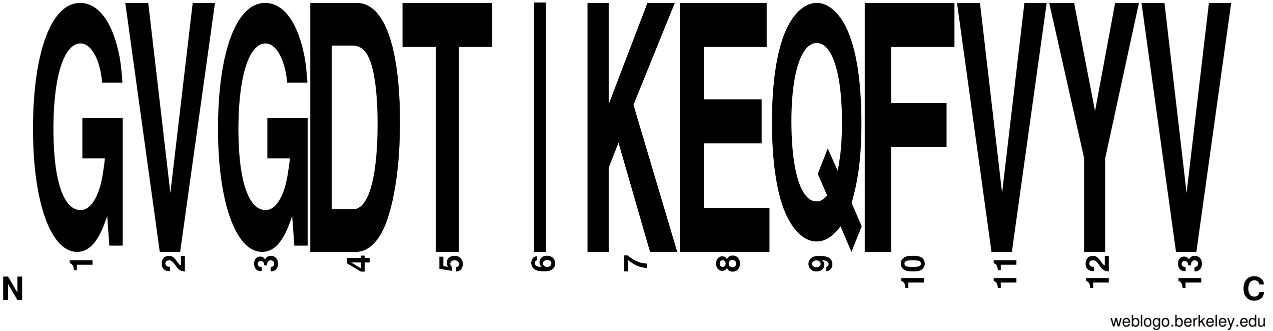
GVGDTIKEQFVYV
1 2 3 4 5 6 7 8 9 10 11 12 13
N
C
weblogo.berkeley.edu

L3-13-III

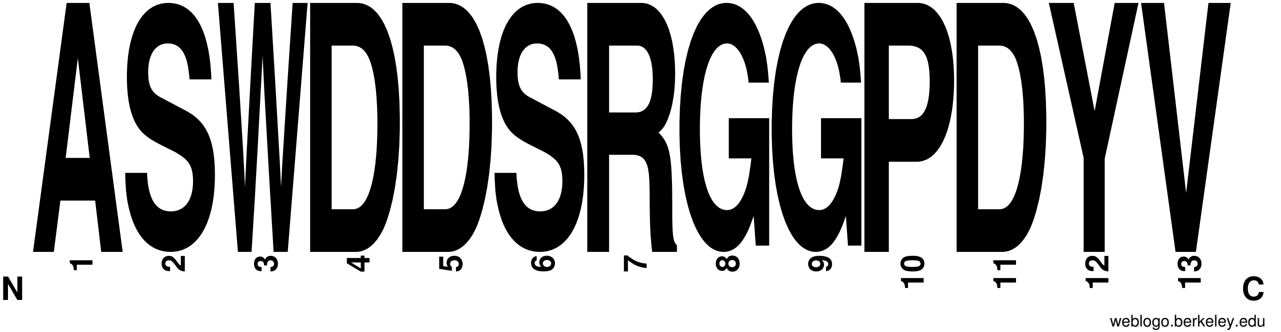
ASWDDSRGGPDYV
1 2 3 4 5 6 7 8 9 10 11 12 13
N
C
weblogo.berkeley.edu
