# Supplementary material for: Disjoint combinations profiling (DCP): a new method for the prediction of antibody CDR conformation from sequence
Source: PeerJ. 2014 Jul 1;2:e455. doi: 10.7717/peerj.455 (PMC4103075; doi:10.7717/peerj.455)
Supplement: Supplemental Information 5 — Canonical templates were derived from the clustering set for every applicable conformational cluster, using the definitions of structurally-determining residues described in Martin & Thornton (1996). [file peerj-02-455-s005.doc]

**Supplementary tables**

**Detailed canonical templates by CDR/Length.**

| **L1/11-residues** | **[L1-11-I]** | **[L1-11-II]** | **[L1-11-III]** | **[L1-11-IV]** |
| --- | --- | --- | --- | --- |
| L2 | I, L, E, C, V, T, N, S, - | Y, I, S | Y | I |
| L4 | M, L, H, V, I | L, V | L | M |
| L25 | A, T, G, E, P, S | G | G, A | A |
| L26 | S, G, T, N | N, E, D | D, E, N | S |
| L28 | N, D, E, S, G, T, V, I, Y, R | I, L | L | A |
| L29 | V, I, T, F, L | G, S, A | P, S, D, G | S |
| L30 | G, K, S, H, R, Y, I, N, D, V, T, E, Q, A | S, D, G, N, R | K, N, D, E | Y |
| L33 | V, I, L, M | V | A, V | V |
| L34 | A, G, N, S, I, H, T, F, V, Y, D, E | H, C, S, I, Q | Y, S | A |
| L36 | Y, L, F, I | Y, N | F, Y | Y |
| L46 | L, T, R, F, V, G, Q, K, S, A, P, I | L, V | L, M | L |
| L49 | Y, H, K, R, F, S, E | Y, S, C | Y | Y |
| L51 | A, T, G, P, V, S | D | D | A |
| L71 | F, Y | A | A, V | F |
| L90 | Q, H, N, S, R, E, G, A | V, T, S | S, A | S |
| L93 | S, N, T, E, G, D, Q, A, R, K, I, F, L, V, Y, M, P, W | S, M, G | G, S, A, N | S |

| **L1/12-residues** | **[L1-12-I]** | **[L1-12-II]** | **[L1-12-III]** | **[L1-12-IV]** |
| --- | --- | --- | --- | --- |
| L2 | I, N, T | I, L | I | L |
| L4 | L | L, M | L, M | L |
| L25 | A, V | A | S, A | L |
| L29 | V, I, F, L | V | I, L | H |
| L33 | L, V, F | L, V | V, L | I |
| L71 | Y, F | F | F, Y | R |
| L90 | Q, K | Q | V, Q | V |
| L91 | Y, W, F, I, G | Y | Y, F | G |
| L93 | R, S, T, G, N, D | S, T, Q, D | A, H | T |

| **L1/13-residues** | **[L1-13-I]** | **[L1-13-II]** | **[L1-13-III]** |
| --- | --- | --- | --- |
| L4 | L | L | L |
| L25 | G | R | G |
| L29 | N, D | N, S | N |
| L30 | I, V | I, L | V |
| L33 | V | V | A |
| L66 | K, R | R, I, V | R |
| L71 | A | A, N | A |

| **L1/14-residues** | **[L1-14-I]** | **[L1-14-II, L1-14-VII]** | **[L1-14-III, L1-14-IV]** | **[L1-14-V]** | **[L1-14-VI]** |
| --- | --- | --- | --- | --- | --- |
| L4 | V | L | L | L | L |
| L25 | S | G | G | G | L |
| L29 | A, T | D, N | I | N | H |
| L30 | V, I | V | V | I | D |
| L31 | N, H | N, D, E | N | F, Y, H | Y |
| L33 | A | V, I | V | V | V |
| L66 | L | K | K | K | K |
| L71 | A | A | A | A | G |
| L90 | L | S | S | S | M |

| **L1/15-residues** | **[L1-15-I]** | **[L1-15-II]** |
| --- | --- | --- |
| L2 | I, M, L, T, N, - | L, I |
| L4 | M, L | L |
| L24 | R, K, G | R |
| L25 | A, S | A |
| L26 | S, N, G | S |
| L28 | S, G, R | S |
| L29 | V, I | V |
| L30 | D, Y, S, E, R, V, T | S |
| L30c | G, T, V, Y | G |
| L33 | M, L, I | M |
| L34 | H, N, S, Q, D, E, Y, A | H |
| L51 | A, S, V, I | A |
| L71 | F | F |
| L90 | Q, Y, H | Y, H |
| L92 | N, K, H, R, W, I, Y | R |
| L93 | E, K, V, Y, G, R | E |

| **L1/16-residues** | **[L1-16-I]** | **[L1-16-II]** | **[L1-16-III]** | **[L1-16-IV]** | **[L1-16-V]** |
| --- | --- | --- | --- | --- | --- |
| L2 | I, L, V, A | I | L | V | V |
| L4 | L, M, V, I | L | M | V | M |
| L25 | S, P, A, F, C | S | S | S | S |
| L26 | S, T, N, G | S | S | S | S |
| L27 | K, Q, R, H | Q | Q | E | Q |
| L29 | L, I | L | L | I | L |
| L30a | N, H, D, Y, K, L | S | H | T | H |
| L30b | S, N, E, T, K, I, A, G | N | S | R | S |
| L30c | N, D, S, T | N | Y | N | S |
| L30d | G, A | R | G | G | G |
| L32 | H, Y, F, S | Y | F | P | Y |
| L33 | M, L, F | L | L | I | L |
| L34 | Y, H, E, I, N, R, F, D, S, Q, A | H | N | E | H |
| L51 | M, V, A, G, I, L | V | V | A | V |
| L71 | F | F | F | F | F |
| L90 | Q, H | Q | Q | D | Q |
| L92 | L, T, S, V, A | S | T | S | T |
| L93 | E, H, L, D, R | H | H | T | H |

| **L3/8-residues** | **[L3-8-I, L3-8-II, L3-8-III, L3-8-V]** | **[L3-8-IV]** | **[L3-8-VI]** |
| --- | --- | --- | --- |
| L36 | Y, F | Y | Y |
| L89 | L, K, H, Y, Q, G, S, M, F | Q | Q |
| L90 | Q, S, H | Q, H | N |
| L91 | Y, S, Q, R, T, F, W, G, H | A, H | W |
| L94 | L, N, Y, G, T, V, F, D, R, P, S | F, T | S |
| L95 | -, P | - | P |
| L97 | T, S | T | T |
| L98 | F | F | F |

| **L3/9-residues** | **[L3-9-I]** | **[L3-9-II]** | **[L3-9-III]** | **[L3-9-IV]** | **[L3-9-V]** | **[L3-9-VI]** |
| --- | --- | --- | --- | --- | --- | --- |
| L2 | I, L, S, T, V, E, M, A, C, N, - | A, N, I, F, L, T, S, - | I, V, L, F, Y | Y | I | - |
| L3 | V, E, Q, K, L, A, T, M | V, M, L, Q, E | A, V, M, Q, E | D | V | E |
| L4 | M, L, V, H, I | V, L, M | L, M | L | L | L |
| L28 | N, S, D, E, G, T, I, R, V, A, Q, Y | G, S, D, R | S, G, L | L | S | S |
| L30 | G, S, H, L, V, -, D, R, I, N, Y, F, K, T, E, Q, P, A | V, S, I, K, - | S, E, -, F, L, R, N | D | R | G |
| L31 | T, S, N, I, K, G, D, H, V, R, Y, L | N, T, H, R, K | S, T, N, Y, R, Q | K | R | R |
| L32 | A, N, Y, H, F, K, T, S, D, W, R, G, P, L, I | Y, H, K, F, N | Y, W, S, R | Y | R | N |
| L33 | V, L, M, I, F | A, L, V, I, M | L, V, M, A | V | V | L |
| L89 | Q, H, A, S, M, L, G, F, V, W, D, K | A, Q, L, W | Q, K | Q | Q | Q |
| L90 | Q, H, N, D, R, E | L, Q, S | K, H, Q, T, V, A | A | V | A |
| L91 | Y, D, F, N, S, V, W, H, G, A, C, T, R, I, L, M | W, Y, F, R | Y, W, S | W | Y | R |
| L92 | S, H, F, W, L, Y, T, N, K, G, A, Q, D, I, V, E, R | Y, S, F, C, D, N, A | G, A, T, Y, D, S | D | G | L |
| L93 | S, R, T, E, N, H, D, G, Q, A, K, I, F, L, V, M, Y, W, P | S, G, N, A, H, I, R | T, G, Y, P, S, A, N | A | A | L |
| L94 | Y, S, L, T, V, H, D, K, F, R, A, W, N, I, G, P, E | N, G, Y, R, S, F, H | S, Y, P, H | S | S | L |
| L95 | P, S, F, Q, L, M | H, P, L, Q, N, K, V, T, I, Y | A, S, L, M, I | T | S | P |
| L96 | L, Y, F, R, P, W, H, I, Q, S, V, T, N, M, A | W, L, F, V, C, Q, Y, R | I, A, Y, V, F, S | G | Y | Q |
| L97 | T, S, K, A | V, T, I | T, V, I | V | T | T |
| L98 | F, V, S | F | F | S | F | F |
| H47 | W, -, L, Y, R, F | W, -, Y, M | W | W | W | W |

| **L3/10-residues** | **[L3-10-I]** | **[L3-10-II, L3-10-XII, L3-10-III, L3-10-V, L3-10-VII]** | **[L3-10-IV]** | **[L3-10-VI]** | **[L3-10-VIII]** | **[L3-10-IX]** | **[L3-10-X]** | **[L3-10-XI]** |
| --- | --- | --- | --- | --- | --- | --- | --- | --- |
| L4 | L | L, V | M, L | L | L | L | M | L |
| L32 | S | Y, S, T, F | Y | M | Y | Y | Y | Y |
| L36 | Y | Y, F | Y | Y | Y | Y | Y | Y |
| L89 | S, K | S, Q, M, G | S, Q | A | Y | Q | Q | L |
| L90 | S | S, V, T, A | Q | T | S | H | Q | Y |
| L91 | L | Y, W, A, L | S, R | W | D | H | H | S |
| L92 | T | E, D, L, V | T, S | D | I | Y | Q | R |
| L95a | H, R | N, S, H, D, G | P | R | Y | P | L | P |
| L96 | R | F, V, A, P, Y, N | L, V, I, W | T | P | L | T | W |
| L97 | I, V | V, R, I | T | V | L | T | T | T |
| L98 | F | F | F | F | F | F | F | F |
| H47 | W, - | -, W, Y | W | Y | W | W | W | W |

| **H1/13-residues** | **[H1-13-I, H1-13-XI]** | **[H1-13-II, H1-13-IV, H1-13-V]** | **[H1-13-III]** | **[H1-13-VI]** | **[H1-13-VII]** | **[H1-13-VIII]** | **[H1-13-IX]** | **[H1-13-X]** |
| --- | --- | --- | --- | --- | --- | --- | --- | --- |
| H2 | V, I, D, -, Q, G, M, L, A, E | V, E | V | V | V | V | V | V |
| H4 | L, X, V, F, -, Q | L | L, M | L | L | L | L | L |
| H20 | I, M, L, V | L, I | L | L | L | L | V | L |
| H24 | A, T, V, G, S | A, V | A | A | A | A | A | A |
| H26 | G, N, D | G, R | G | R | G | G | G | G |
| H29 | F, S, Y, I | F, G, V, S, Y, L | Y, I, W, H | G | G | Y, F, A, D | F | D |
| H32 | H, Y, S, F, D, A, N, C, V, R, E, Q | Y, D, G, N, W, C, F | I, Y | W | Y | K, N, E | Y | Y |
| H33 | V, N, W, Y, A, T, G, E, S, F, D, L, R, Q, I, P, M, H, K | W, D, Y, T, A, N | Y, C | C | D | Y, D, S | A | C |
| H34 | I, M, L, V, F, W, H | M, I, W, T, L | M | M | M | M, L | I | M |
| H35 | H, Y, G, N, S, E, D, K, Q, T, F, A, L, W, V, R | S, G, H, A, D, N | G | G | G | G, T, D | S | A |
| H48 | I, L, V, M | V, I, M | V | V | V | V | M | V |
| H51 | I, F, V, N, S, M, K, L, T, A | I, V | M, I | L | I | I, V, L | I | I |
| H69 | L, I, F, M, V, T, S | I, L | I | V | I | I, V | I | F |
| H78 | A, L, F, V, G, I, T, Y, M | L, V, A | V | V | V | V, A | V | V |
| H80 | M, L, V, I, H, Q | L, M | L | L | L | L | M | L |
| H90 | Y, F | Y | Y | Y | Y | Y | Y | Y |
| H94 | R, K, I, H, G, T, N, S, M, A, P, L | L, A, R, Y, V, I | A | A | A | A | K | T |
| H102 | Y, F, I, H, V, S, G, D, N, R, L, P, T, K, M, W | Y, S, V, I | Q, S, R | G | W | S, Y | V | Y |

| **H1/15-residues** | **[H1-15-I]** | **[H1-15-II]** | **[H1-15-III]** |
| --- | --- | --- | --- |
| H20 | L, I | L | L |
| H24 | V, F, L, I, A | F | A |
| H26 | G | G | G |
| H28 | S | S | R |
| H29 | I, L, V, T | L, I | F |
| H34 | W, V | V | M |
| H48 | M, L, I, V | L | V |
| H53 | Y, W, S, N | W | S |
| H78 | F, V, A, L | V | L |
| H80 | I, L | L, M | L |
| H94 | R, Q, H, L | R | S |

| **H2/9-residues** | **[H2-9-I, H2-9-VI, H2-9-VII]** | **[H2-9-II]** | **[H2-9-III]** | **[H2-9-IV]** | **[H2-9-V]** |
| --- | --- | --- | --- | --- | --- |
| H47 | W, Y, G, L, F | W | W | G, W | W |
| H51 | I, M, V | I | A | I, V | I |
| H55 | G, D, A, E | F, G, V | V | S | G |
| H59 | Y, F, H, L | Y, G | Y | G, N | Y |
| H69 | I, M, L, V, F, T | I | I | S, T | I |
| H71 | K, R, V, Q, M, I | S, V, Q | V | D | R |

| **H2/10-residues** | **[H2-10-I]** | **[H2-10-II, H2-10-III, H2-10-IV, H2-10-V, H2-10-VI, H2-10-IX]** | **[H2-10-VII]** | **[H2-10-VIII]** | **[H2-10-X]** |
| --- | --- | --- | --- | --- | --- |
| H33 | Y, W, F, E, C, T, N, L, V, G, P, -, D, A, S, R, I, H | A, T, G, Y, D, H, V, W, F, S, R, C, Q, I, P, M, K | Y, C | Y | D |
| H47 | W, G, L, S, Y | W, S, I, F, L, G, Y | L, G | W | W |
| H50 | W, Q, R, E, G, L, N, A, Y, D, V, S, T, M, I, H, F | G, F, S, T, A, Y, L, V, E, I, D, N, Q, R, M, W, K | A, F | W | W |
| H51 | I, V, L, F, N, K | I, V, M, L, T, F | I | I | I |
| H52 | D, H, L, Y, N, V, I, F, R, S, A, T, E, K | S, N, F, R, T, W, H, G, D, A, K, V | N | F | Y |
| H53 | E, A, D, N, G, S, Y, K, T, R, H, I, F, M, L | N, D, G, S, V, A, R, T, L, E | D | G | G |
| H54 | I, N, S, G, D, T, Q, Y, R, F, A, V, H, W | T, G, E, S, D, Y, R, A | G | S | D |
| H55 | G, D, S, Y, R, A, V, N | G, S, T, D, A, Y, V, K, R, L, E, N, I, M | E, G | D | G |
| H56 | D, N, G, Y, S, R, I, E, A, T, H, F, K | T, K, R, N, Y, S, I, E, D, G, L, A, P, M | P, I | N | S |
| H58 | E, K, S, N, H, A, V, Y, L, G, T, D, R, I, F, M, Q | I, Y, H, N, D, G, S, F, W, L, R, A, K, Q, T | Y | E | M |
| H59 | Y, F, S, N, L, H | Y, S, H, F, L | Y | Y | Y |
| H69 | M, I, L, F, V | I, L, V, T, S, F, M | I | L | L |
| H71 | A, V, Q, S, L, T, R | R, D, A, V, Q, K, E | R, Q | A | A |
| H78 | A, V, L, G, T | L, V, A, M, G | L, V | A | A |

| **H2/12-residues** | **[H2-12-I]** | **[H2-12-II]** | **[H2-12-III]** | **[H2-12-IV]** |
| --- | --- | --- | --- | --- |
| L94 | K, V, R, L, Y, D, G, N, T, S, F | Y | H | S |
| H47 | W | W | W | W |
| H50 | F, E, R, Q, A, D, L, S | E | F | L |
| H51 | I, S, F | I | I | I |
| H54 | G, N, K, S | N | D | T |
| H55 | Y, F, H, G, D | F | Y | Y |
| H59 | Y | Y | Y | Y |
| H69 | I, M, V | I | I | I |
| H71 | R, V | R | R | K |
| H78 | L, F, V, I, M, A | L | L | V |
